# Comparative 3D‐anatomy of Appendicularian Endostyles (Tunicata, Chordata) ‐ A Tale of Reduction

**DOI:** 10.1002/jmor.70061

**Published:** 2025-06-28

**Authors:** Mai‐Lee Van Le, Seowon Park, Thomas Stach

**Affiliations:** ^1^ Institute of Biology, Comparative Electron Microscopy Humboldt University Berlin Berlin Germany

**Keywords:** Chordate Evolution, Larvacea, Oikopleura, Thyroid Homolog, Urochordata

## Abstract

Appendicularia comprises about 70 holoplanktonic species traditionally classified in three families: Oikopleuridae, Fritillariidae, Kowalevskiidae. Despite their eminent phylogenetic position and their important role in ocean ecosystems, most research focuses on the model organism *Oikopleura dioica* while the diversity of appendicularians remains underexplored. Here, we present a comparative morphological analysis of appendicularian endostyles, a pharyngeal gland homologous to the vertebrate thyroid. Based on light‐ and transmission electron‐microscopical investigations in 12 species representing seven (of 15) genera from all three family‐level taxa, we describe the 3D‐anatomy of endostyles, histologically recognizable cell‐types, and discuss our findings in a cladistic framework. We identified seven different cell types arranged in species specific patterns, including the formerly unrecognized ‘bright cells’. Two ciliary bands – the peripharyngeal band and the retropharyngeal band – are associated with the endostyles. Outgroup comparison indicates that repeated apomorphic reductions of cell types, rows of cells, the retropharyngeal band, and the complete endostyle occurred within Appendicularia. We propose a phylogenetic hypothesis that suggests that “Oikopleuridae” is a paraphyletic grouping and supports an evolutionary scenario with multiple reductions functionally related to the evolution of the external filter house of appendicularians. While we document the diversity of endostyle anatomy, more detailed cladistic analysis, including other organ systems, is needed to resolve the phylogenetic relationships and to understand the evolution of appendicularian taxa.

## Graphical abstract

Appendicularia are chordates that possess endostyles, a pharyngeal gland homologous to vertebrate thyroid. Here, we describe the detailed 3D‐anatomy of endostyles based on light‐ and electron microscopy. We identified seven cell types arranged in specific patterns. We propose a phylogenetic hypothesis that suggests an evolutionary scenario with multiple reductions functionally related to the evolution of the external filter house of appendicularians.

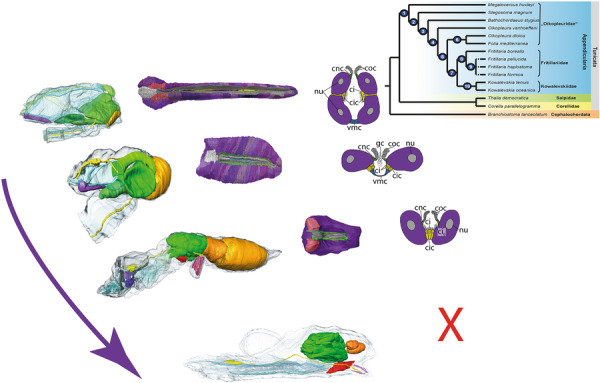

## 1 Introduction

The endostyle, a multifunctional organ in the pharynx, is one of the unambiguous apomorphic traits that characterizes Chordata as a monophyletic group (Ruppert 1997a, Stach 2008). Glandular cells in the endostyle produce an intricate mucous net that is propelled with locomotory cilia into the pharynx (Flood and Fiala‐Medioni 1981; Holley 1986; Nash 2002). This net is used in feeding by internally filtering food particles out of the water. In addition, the endostyle probably functions as an endocrine organ and regulates metamorphosis by producing iodinated signal proteins that belong to the thyroid hormones (Barrington 1962; Paris et al. 2010). In marine invertebrate chordates, i.e., tunicates and cephalochordates, the endostyle forms a ventral groove along the pharynx. Ammocoetes, the larvae of the jawless fish‐like lamprey, have an endostyle which metamorphoses into the thyroid gland of the adult lamprey (Richardson et al. 2010; Manzon and Manzon 2017). The similarities in anatomical position, histological composition, and function support the hypothesis that the endostyle is the homolog of the vertebrate thyroid. This hypothesis has been suggested early on (Müller 1873; Kieckebusch 1928; Barrington 1962; Wright and Youson 1976, 1977, 1980). It is now also supported by investigations of gene expression (Ogasawara et al. 1999; Ogasawara 2000; Hiruta et al. 2005; Onuma et al. 2021; Takagi et al. 2022) and single cell RNA‐sequencing (Grau‐Bové et al. 2024; Jiang et al. 2023, 2024), which demonstrated similarities between tunicate and cephalochordate endostyles and vertebrate thyroids on molecular levels. Most research has emphasized the functional aspects and the evolutionary transition from invertebrate chordate endostyle to vertebrate thyroid with a focus on a few selected model species, mainly the ascidian *Ciona robusta* (Satoh et al. 2003), the cephalochordate *Branchiostoma floridae* (Holland et al. 2004), the lamprey *Petromyzon marinus* (Nikitina et al. 2009), and, more recently, the appendicularian *Oikopleura dioica* (Ferrández‐Roldán et al. 2019).

Early microscopists (e.g., Salensky 1903, 1904; Ihle 1907, 1908) have noticed the diversity of endostyle morphology and have used it as a means to suggest evolutionary trends within Appendicularia, a group of tunicates. Lohmann (1933) e.g., suggested that a rod‐like endostyle was characteristic for Oikopleuridae, one of the three families in Appendicularia, whereas a compressed and dorsoventrally curved shape was found in the family Fritillariidae. The endostyle is entirely absent in the third family Kowalevskiidae, indicating evolutionary reduction. These authors produced pioneering microscopy results and documented a surprising morphological disparity and complexity, confirmed for the model species *O. dioica* (Olsson 1965). Nevertheless, their studies were hampered by difficulties in fixation quality as admitted by Ihle (1908) and naturally lacked a cladistic framework and modern morphological techniques such as high‐resolution microscopy based on semithin sections and digital 3D‐reconstructions. In any case, the morphological disparity of the endostyles hints at its high potential as an indicator of evolutionary history across a wider range of species. In an investigation of the molecular developmental architecture, Cañestro et al. (2008) and later on Onuma et al. (2021) could document that the endostyle of the appendicularian model species *O. dioica* is indeed patterned in a complex manner on the molecular level as well. A reinvestigation using adequate morphological techniques is therefore needed.

Appendicularia consists of about 70 marine planktonic species with a simple tadpole‐like anatomy characterized by a trunk and a locomotory tail (Lohmann 1933; Fenaux 1998a; Hopcroft 2005; Bucklin et al. 2021). Although the animals consist of only few organ systems, their stereotypic and complex epithelium produces a surprisingly intricate mucus house, with which they concentrate food particles before ingesting them (Körner 1952; Flood 1994; Conley et al. 2018; Razghandi et al. 2021). After ingestion, the particles are captured in an inner mucus net produced by the ventral, pharyngeal endostyle (Lohmann 1933; Fenaux 1998a). Ecological studies have demonstrated the enormous effect of appendicularians in the world's oceans, such as carbon sequestration and nutrient provision into deep ocean layers from shedding of their houses (Alldredge et al. 2005; Deibel et al. 2005; Katija et al. 2017). Despite their evolutionary and ecological significance, most appendicularian research has been limited to the globally abundant model species *Oikopleura dioica*. The insightful research on Appendicularia by previous workers, summarized in the “Handbuch der Zoologie” by Lohmann (1933), provides an excellent starting point for more rigorous morphological studies of other appendicularian species.

Here, we present an analysis of the morphological disparity of endostyles based on light‐ and electron‐microscopical investigation of endostyles in 12 species representing seven (of 15) genera from all three traditionally recognized family‐level taxa, Oikopleuridae, Fritillariidae, and Kowalevskiidae. We describe the 3D‐anatomy of the organ, histologically distinguishable cell‐types, and discuss our findings in a cladistic paradigm.

## 2 Methods

The morphology of 12 appendicularian species was analyzed (see Table 1). Specimens of *Stegosoma magnum*, *Folia mediterranea*, *Fritillaria formica*, *Fritillaria haplostoma*, *Kowalevskia tenuis* and *Kowalevskia oceanica* were provided from the appendicularian collection of Dr. Rade Garic at the Institute for Marine and Coastal Research (Institut za more i priobalje ‐ Dubrovnik, Croatia). These specimens were collected via plankton nets by oblique tows between the island Lokrum and mainland Dubrovnik. Specimens were kept for 1–11 years in 10% buffered Formaldehyde and transported to the Laboratory of Comparative Electron Microscopy at Humboldt University Berlin (Germany) for further preparation.

**Table 1:** Information on specimens analyzed in the present study. For additional details see Materials & Methods section.

	Higher Taxon	Origin	Fixation	Preservation	Quality of fixation
*Stegosoma magnum* (Langerhans, 1880)	Oikopleuridae	IZMP & MfNB (ZMB Tun 1611)	bFA &?OsO_4_	bFA & EtOH	+/−
*Folia mediterranea* (Lohmann, 1899)	Oikopleuridae	IZMP	bFA	bFA	—
*Fritillaria formica* Fol, 1872	Fritillariidae	IZMP	bFA	bFA	+/−
*Fritillaria haplostoma* Fol, 1872	Fritillariidae	IZMP	bFA	bFA	+/−
*Kowalevskia tenuis* Fol, 1872	Kowalevskiidae	IZMP	bFA	bFA	+/−
*Kowalevskia oceanica* Lohmann, 1899	Kowalevskiidae	IZMP	bFA	bFA	+/−
*Oikopleura vanhoeffeni* Lohmann, 1896	Oikopleuridae	MfNB (ZMB Tun 1187)	?OsO_4_	EtOH	+
*Megalocercus huxleyi* (Ritter in Ritter & Byxbee, 1905)	Oikopleuridae	MfNB (ZMB Tun 1608)	?OsO_4_	EtOH	++
*Bathochordaeus stygius* Garstang, 1937	Oikopleuridae	MBARI	GA => OsO_4_	n.a.	+++
*Oikopleura dioica* Fol, 1872	Oikopleuridae	M‐LVL & TS @ Villefranche sur mer	GA + OsO_4_	n.a.	+++
*Fritillaria pellucida* (Busch, 1851)	Fritillariidae	M‐LVL & TS @ Villefranche sur mer	GA => OsO_4_	n.a.	+++
*Fritillaria borealis* Lohmann, 1896	Fritillariidae	SARS	GA => OsO_4_	n.a.	++

Abbreviations: IZMP, Institut za more i priobalje courtesy of Dr. Rade Garic; MfNB, Museum für Naturkunde Berlin (collection number: ZMB Tun XXXX); MBARI, Monterrrey Bay Aquarium and Research Institute courtesy of Dr. Rob E. Sherlock; M‐LVL & TS @ Villefranche sur mer, collected and fixed by Mai‐Lee Van Le and Dr. Thomas Stach at the Observatoire Oceanologique de Villefranche; SARS, Michael Sars Centre courtesy of Dr. Simon Henriet and Anne Aasjord; bFA, unknown duration in 10% buffered formaldehyde; GA => OsO_4_, primary and secondary fixation (see Materials & Methods section) interrupted by express transport in buffer for up to 3 days; GA + OsO_4_, primary and secondary fixation (see Materials & Methods section) carried out consecutively; EtOH, storage in 70%EtOH;?OsO_4_, unknown concentration and unknown duration in Osmiumtetroxide‐solution; Quality of fixation, very good (+++), good (++), adequate (+), acceptable (+/−), deficient but recognizable (−).

Specimens of *Stegosoma magnum*, *Oikopleura vanhoeffeni* and *Megalocercus huxleyi* were provided by the Natural History Museum (Museum für Naturkunde ‐ Berlin, Germany). These specimens were previously treated with Osmiumtetroxide (OsO_4_) and preserved in 70% ethanol. Unfortunately, the original records of the collections could not be located at the museum. Therefore, details about the fixation remain unknown.

Two specimens of *Bathochordaeus stygius* were provided by the MBARI (Monterey Bay Aquarium Research Institute; California USA). They were collected via the remotely operated vehicle Ventana in the Monterey Bay (California USA) using gentle suction (Robison 1993).

Animals were maintained alive until they could be fixed onshore using a solution of 1% paraformaldehyde, 2.5% glutaraldehyde in 0.2 mol l^‐1^ sodium cacodylate buffer (pH 7.2), and adjusted to an osmolarity of approximately 800 mOsm with the addition of sodium chloride. Primary fixation was stopped after 1 h with three buffer rinses. Animals were stored in the same buffer and shipped in the buffer via express mail to the Laboratory of Comparative Electron Microscopy at Humboldt University (Berlin, Germany) for postfixation.

Specimens of *Oikopleura dioica* and *Fritillaria pellucida* were collected at the Observatoire Océanologique de Villefranche (Villefranche‐sur‐Mer, France) via oblique plankton tows. Animals were fixed in a solution of 1% paraformaldehyde, 2.5% glutaraldehyde in 0.2 mol l^‐1^ sodium cacodylate buffer (pH 7.2) adjusted to an osmolarity of approximately 800 mOsm with added NaCl. After 1 h, primary fixation was stopped with three buffer rinses. Animals were kept in the same buffer and shipped via express mail to the Laboratory of Comparative Electron Microscopy at Humboldt University Berlin (Germany).

Specimens of *Fritillaria borealis* were provided from the appendicularian facility of the SARS centre (Michael Sars Centre ‐ Bergen, Norway). Adult specimens (5–7 days old) were isolated from the culture then immediately fixed in a solution of 1% paraformaldehyde, 2.5% glutaraldehyde in 0.2 mol l^−1^ sodium cacodylate buffer (pH 7.2) adjusted to an osmolarity of approximately 800 mOsm with added NaCl. After 1 h, primary fixation was stopped with three buffer rinses. Animals were kept in the same buffer and shipped via express mail to the Laboratory of Comparative Electron Microscopy at Humboldt University Berlin (Germany).

Postfixation was performed on all specimens in a solution of 1% Osmium tetroxide (OsO_4_) in double‐distilled water (ddH_2_O) and stopped with three rinses (once for 15 min and twice for 30 min) with ddH_2_O. After dehydration through a graded series of ethanol, the specimens were embedded in Araldite and subsequently used for histological analysis. Semithin sectioning of entire trunks of specimens was accomplished via a Leica Ultracut S, resulting in transverse series of all specimens except *Fritillaria borealis* where a second specimen was cut longitudinally. Specimens were cut with 0,5 µm to 1 µm (see supporting table 1) thickness.

Histological sectioning of *Fritillaria borealis* was completed with combined alternating semithin and ultrathin sectioning. The embedded specimens were sectioned from the posterior end towards the anterior end with 1 µm thickness until the esophagus was reached, after which alternating sections were cut with 16 semithin sections (0.5 µm) followed by ca. 30 ultrathin sections (60 nm). This provided section series with an overview of the complete specimens with detailed ultrastructural information in the anterior region of the trunk.

Semithin sections were stained using 0.5% toluidine blue in a solution of 1.5% sodium bicarbonate and 40% glycerol. Ultrathin sections were stained with 2% uranylacetate and 2.5% lead citrate in an automatic stainer (courtesy of Dr. Björn Quast, Universität Bonn and Dr. Alexander Gruhl, MPI Bremen). Stained ultrathin sections were examined under a Zeiss EM9 transmission electron microscope, operated at 80 kV and recorded with a Wide‐angle Dual Speed 2 k‐CCD‐Camera (TRS – Tröndle Restlichtverstärkersysteme, Germany).

Light micrographs were recorded with a Canon EOS 700 D mounted on a Zeiss Axioplan compound microscope to create 3D‐reconstructions in Amira (see supplementary table 1). Reconstructions were manually performed in the software Amira 6.4.0 (Thermo Fisher Scientific), resulting in 3D anatomical models of the animals’ entire trunk, the endostyle and the pharyngeal ciliary bands. Endostyles were reconstructed on a cellular level except for *Fritillaria formica* and *Fritillaria haplostoma*, where specimen size in combination with insufficient fixation quality prevented reconstruction at the cellular level. Trunk anatomy, organ systems, and ciliary bands were reconstructed at the tissue level.

## 3 Results

**Figure 1:**
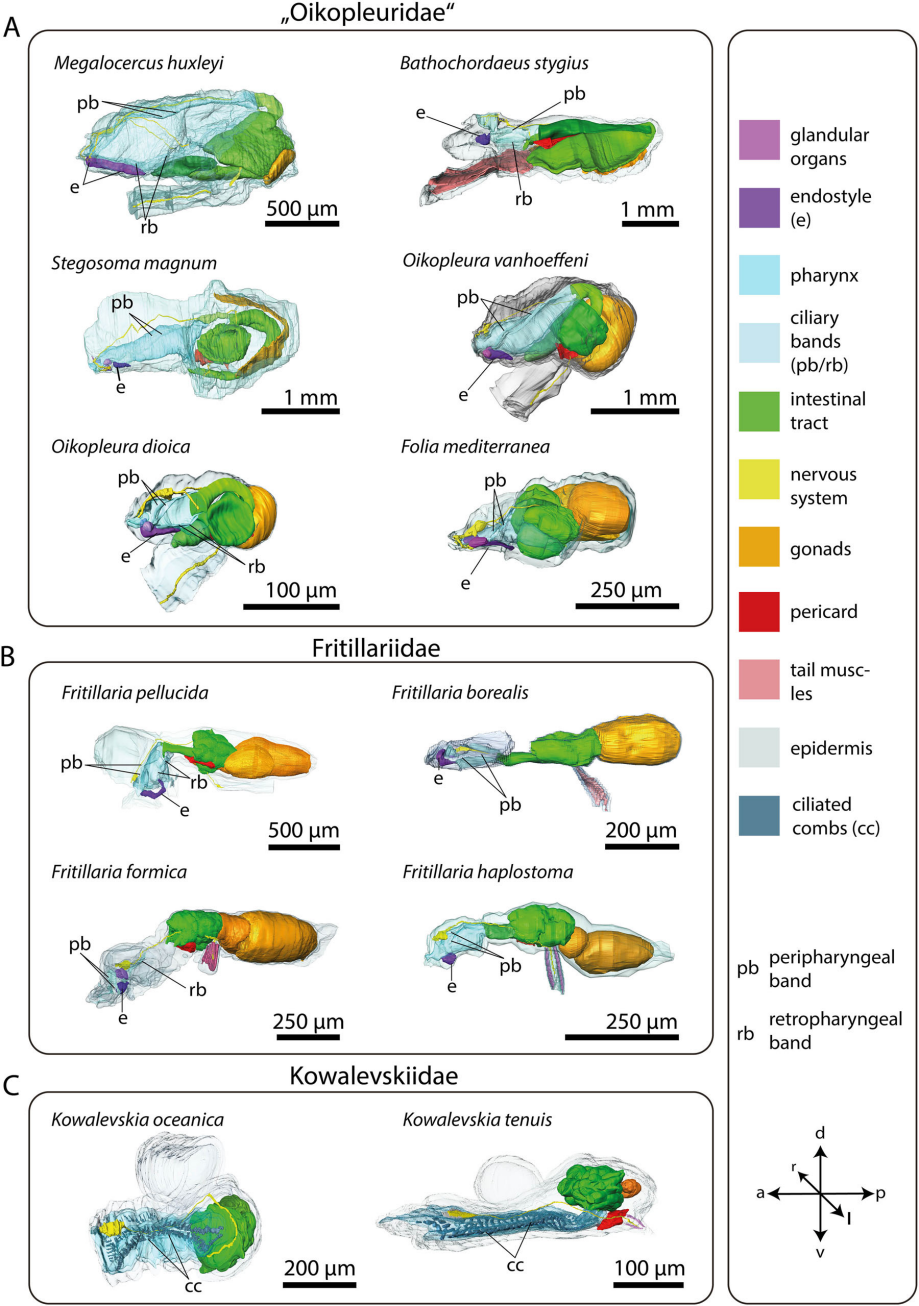
3D‐reconstructions of complete specimens of 12 appendicularian species. (A–C) Tails are partially reconstructed. Note the morphologically distinct form and constriction of the trunk in fritillariids as well as the lack of an endostyle in kowalevskiids.

The pharynx, with lateral gill openings, is located in the anterior portion of the trunk (Figure 1). Where present, the endostyle resides ventrally in the pharynx. 3D‐reconstructions of the entire trunks visualize the anatomy of inner organ systems including the pharynx, the endostyle, and associated ciliary bands (Figure 1). The endostyle, a glandular organ is present in all species analyzed except kowalevskiids, where the endostyle is entirely absent with no remnants of glandular cells (Figure 1C). In addition, the pharynx in Kowalevskiidae is distinct from the pharynges in Oikopleuridae and Fritillariidae; in kowalevskiids, it opens on both sides through spiracles modified into long ciliated fissures and it possesses a unique filtering system consisting of ciliated combs arranged in two pairs of opposing longitudinal rows.

### 3.1 General Endostyle Morphology in Appendicularia

**Figure 2:**
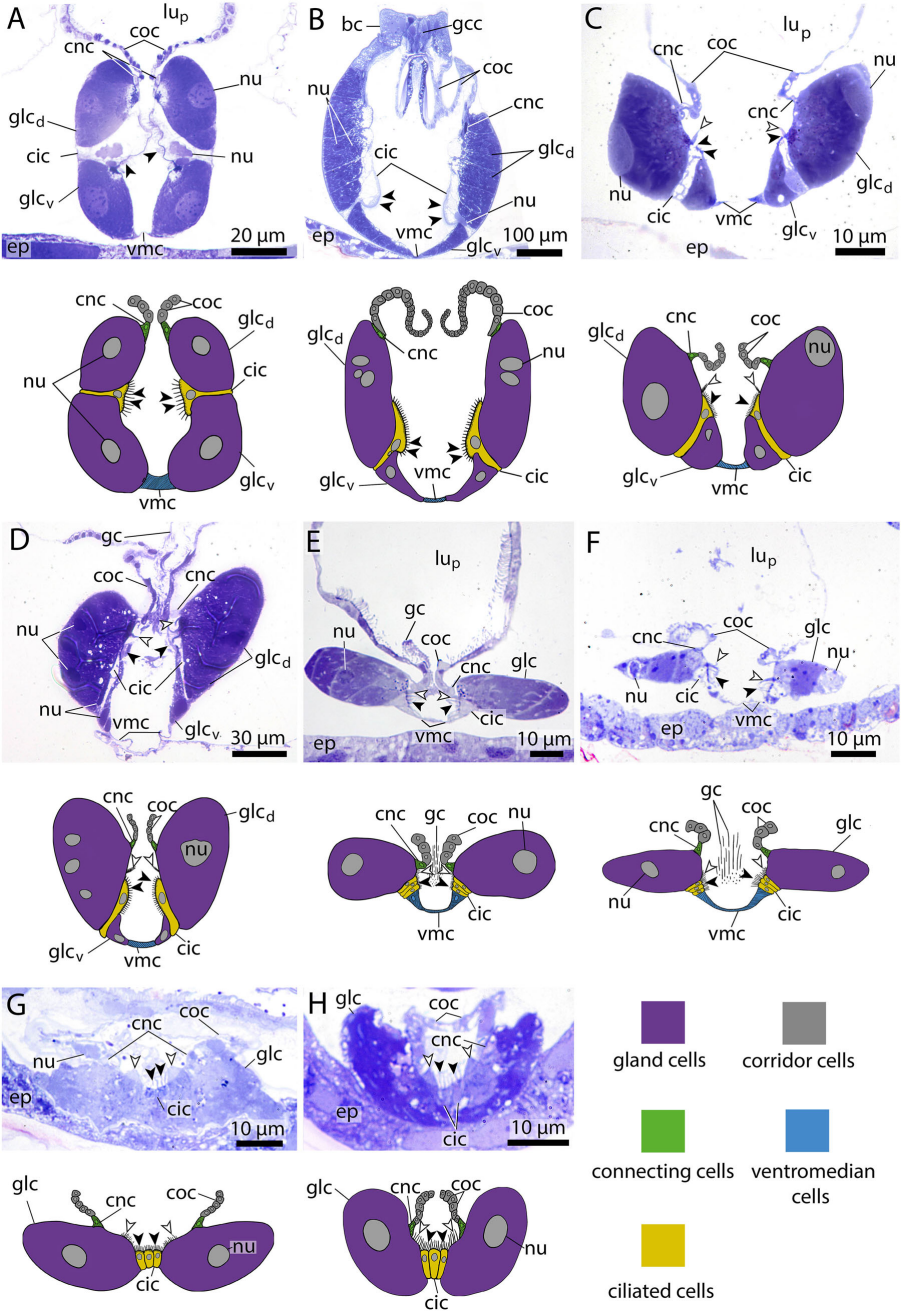
Light micrographs of endostyle cross sections at the approximate center in eight appendicularian species and respective illustrations showing the different cell types present in the analyzed species. Black arrowheads point to cilia from ciliated cells, white arrowheads to cilia from gland cells. (A) *Megalocercus huxleyi*, (B) *Bathochordaeus stygius*, (C) *Stegosoma magnum*, (D) *O. vanhoeffeni*, (E) *Oikopleura dioica*, (F) *Folia mediterranea*, (G) *Fritillaria pellucida*, (H) *Fritillaria borealis*. Note the two paired rows of gland cells (glc) separated by ciliated cells (cic) in *M. huxleyi, B. stygius* and *S. magnum*. No ventromedian cells are present in *F. pellucida* and *F. borealis*. coc – corridor cells, cnc – connecting cells, ci – cilia from ciliated cells, ci_g_ – cilia from gland cells, nu – nuclei, vmc – ventromedian cells, gcc – giant cilia cells, gc – giant cilia, lu_p_ – pharynx lumen, glc_d_, – dorsal gland cells, glc_v_ – ventral gland cells.

In Oikopleuridae and Fritillariidae the endostyle lies in the anterior region of the trunk ventrally in the pharynx. Dorsally, the endostyle is continuously connected with the pharyngeal epithelium and opens into the lumen of the pharynx (Figures 1A,B, 2). In oikopleurids, the opening stretches along the anterior‐posterior axis almost along the complete length of the endostyle (Figure 1A). In *Fritillaria pellucida* the opening is restricted to a small area at the anterior dorsal region of the endostyle. In *Fritillaria borealis* this opening is also extremely narrow.

Seen from the side, the overall shape of the endostyle is rod‐like in oikopleurids and slightly dorsoventrally curved in fritillariids. It is straight, rodlike and simultaneously broad and dorsoventrally flattened in *Oikopleura dioica* and *Folia mediterranea* (Figures 1, 5).

Comparison of length to width ratios, reveals a relatively long endostyle in *M. huxleyi* (l/w = 4.7) and moderately elongated endostyles with a length to width ratio of 2.8 in *F. pellucida* and 2.6 in *S. magnum*. Species with intermediate proportions include *Oikopleura dioica* (2.0), *Bathochordaeus stygius* (1.8), *Folia mediterranea* (1.68), and *Fritillaria borealis* (1.66). The most compact endostyles are found in *Oikopleura vanhoeffeni* (1.5), *Fritillaria haplostoma* (1.45) and *Fritillaria formica* (1.45) (Table 2).

**Table 2:** Characteristics of appendicularian endostyles.

Species	Endostyle	Length/width ratio	Number of gland cell rows	Total number of gland cells	Bright cells present	Giant cilia cells	Ventromedian cells
*Megalocercus huxleyi*	✓	4.7	2	74	✓	✓	✓
*Stegosoma magnum*	✓	2.6	2	80	✓	✓	✓
*Bathochordaeus stygius*	✓	1.8	2	42	✓	✓	✓
*Oikopleura vanhoeffeni*	✓	1.5	1.5	40	✓	✓	✓
*Folia mediterranea*	✓	1.68	1	46	✕	✓	✓
*Oikopleura dioica*	✓	2.0	1	38	✕	✓	✓
*Fritillaria borealis*	✓	1.66	1	24	✓	✕	✕
*Fritillaria pellucida*	✓	2.8	1	18	✕	✕	✕
*Fritillaria haplostoma*	✓	1.45	1	?	?	✕	✕
*Fritillaria formica*	✓	1.46	1	?	?	✕	✕
*Kowalevskia tenuis*	✕	n.a.	✕	n.a.	✕	✕	✕
*Kowalevskia oceanica*	✕	n.a.	✕	n.a.	✕	✕	✕
*Thalia democratica*	✓	> 5	3	> 80	?	✓	✓
*Corella parallelogramma*	✓	> 5	3	> 80	?	✓	✓
*Branchiostoma lanceolatum*	✓	> 5	2	> 80	?	✓	✓

*Note:* ✓ ‐ present, ✕ ‐ absent,? ‐ unknown, n.a. ‐ not applicable. Data on tunicate outgroups are from Fredriksson et al. (1988), on amphioxus from Olsson (1963) and Ruppert (1997b).

### 3.2 Endostyle Cell Types

**Figure 3:**
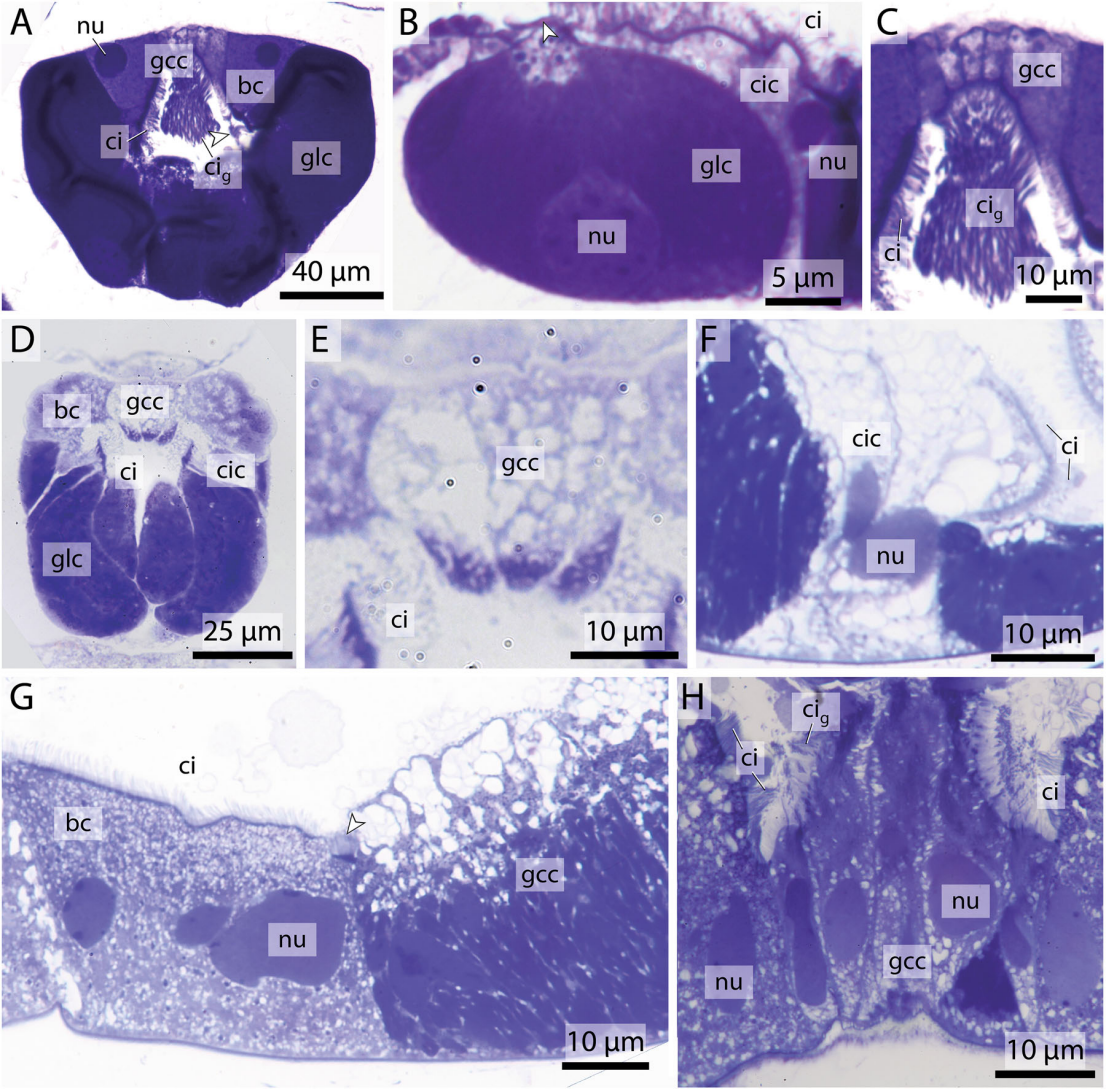
Light micrographs of endostyle cross sections illustrating different cell types. (A–C) *Megalocercus huxleyi*. (D, E) *Stegosoma magnum*. (F–H) *Bathochordaeus stygius*. bc – bright cell, ci – cilia, cic – ciliated cell, ci_g_ – giant cilia, gcc – giant cilia cell, glc – gland cell, nu – nucleus, arrowheads point to cilia on gland cells.

**Figure 4:**
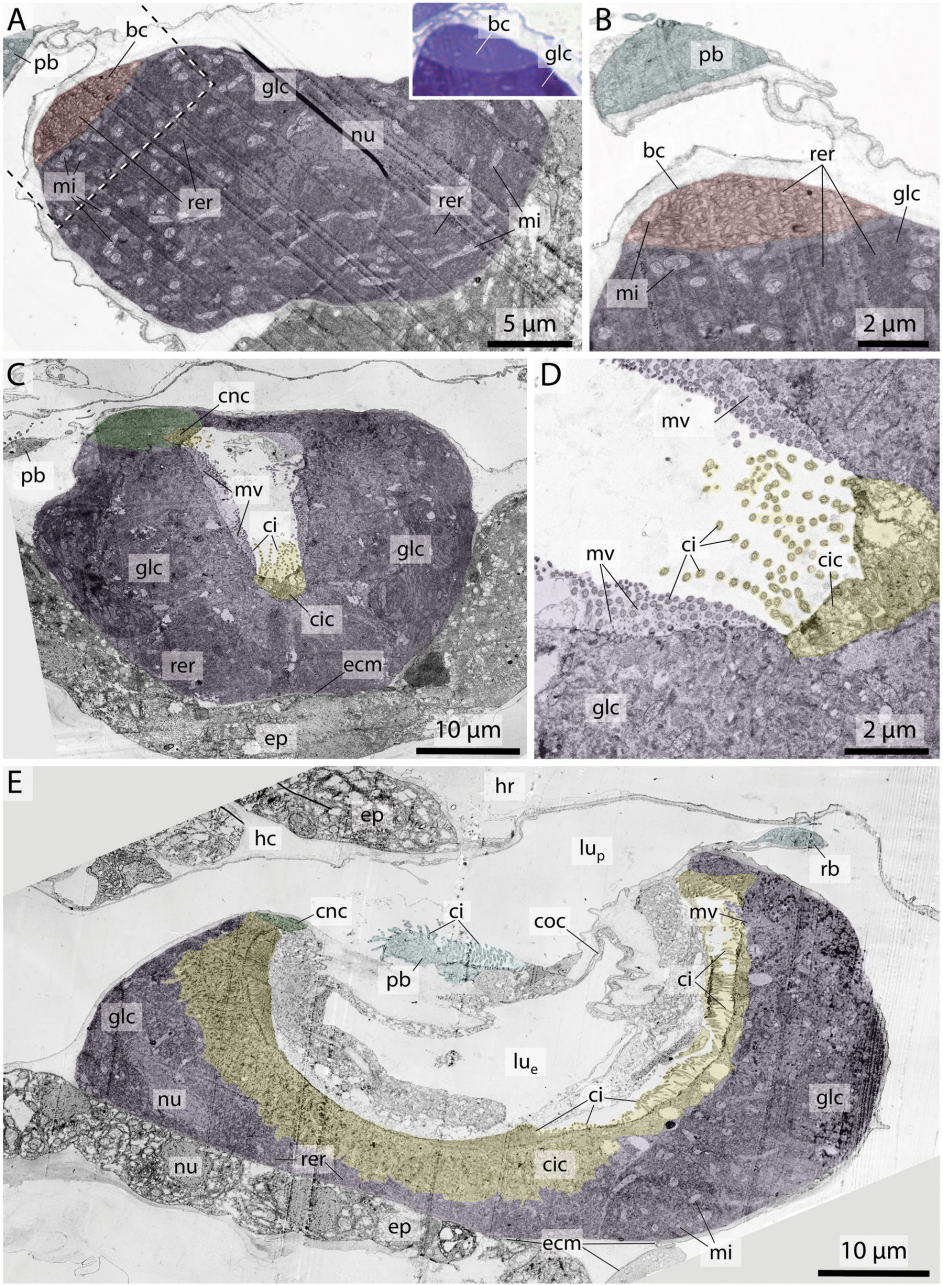
Electron micrographs from the endostyle in *Fritillaria borealis*. (A version of this figure without the transparent coloration can be found as Figure S2 in the supplemental data.) (A) Anterior to the left posterior to the right. Longitudinal section through the left side of the endostyle displaying gland cells (glc) with nuclei (nu) and an adjacent bright cell (bc) with less densely packed endoplasmic reticulum. Inset: light micrograph of an adjacent section showing the difference in toluidine staining between a gland cell and a bright cell. (B) Closeup of area marked by dashed rectangle in A showing the bright cell as well as the adjacent gland cell with mitochondria (mi). Notice the different densities of endoplasmic reticulum. (C) Cross section approximately between the center and the anterior end of the endostyle. Displaying the two paired rows of gland cells with ciliated cells (cic) nested at the ventral inner midline and their cilia (ci). (D) Closeup of C displaying the arrangement of cilia in ciliated cells as well as the densely packed combination of cilia and microvilli from gland cells. (E) Longitudinal section through the approximate center midline of the endostyle, displaying gland cells, ciliated cells with cilia as well as connecting cells (cnc) and corridor cells (coc).

**Figure 5:**
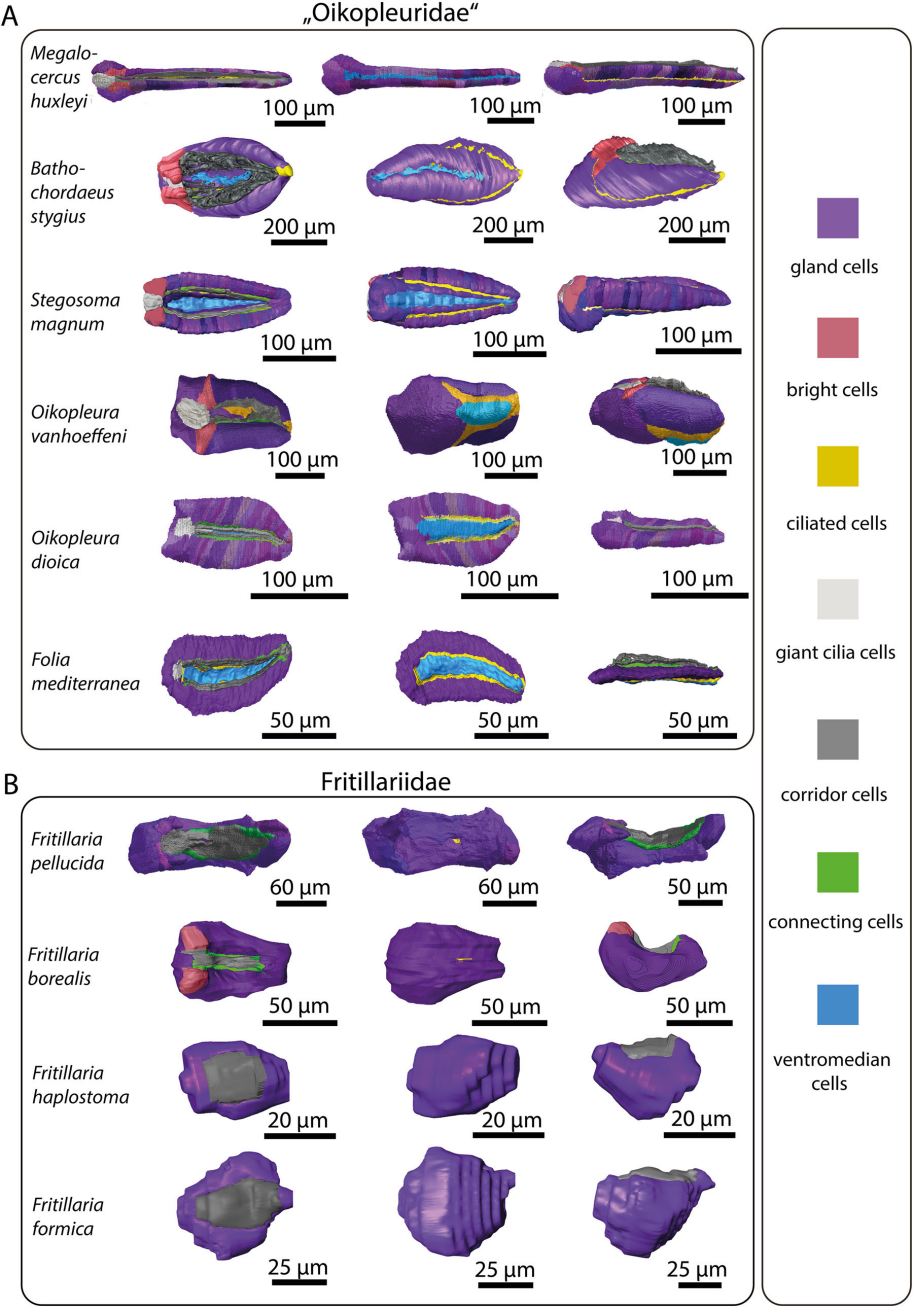
3D‐reconstructions of endostyles in 10 appendicularian species showing general morphology and different cell types. From left to right: dorsal view, ventral view, lateral view. (A) Oikopleurids. (B) Fritillariids. Note the more rod‐shaped appearance of oikopleurid endostyles and the dorsoventrally curved shape of fritillariid endostyles. Giant cilia cells (light grey) and ventromedian cells (blue) are present in oikopleurids and absent in fritillariids. Note: The reconstructions of *Fritillaria haplostoma* and *Fritillaria formica* are of lower resolution but illustrate the overall shape and dimensions of the endostyles. For further details, see Methods.

In the following description of cell types, we use the terminology introduced by Olsson (1965), who studied the ultrastructure of the endostyle in *Oikopleura dioica* and distinguished six cell types. In the present study, we found all six types of cells described by Olsson and identified an additional one. From ventral to dorsal these seven cell types are:

1. **Ventromedian cells** occur exclusively in oikopleurid species and are absent in fritillariids. They are the only cell type bearing no cilia or microvilli and occupy the ventral trough of the endostyle. Ventromedian cells are usually thin, measuring often less than 1 µm in height and stretching over more than 10 µm in cross sections. Only around the nuclei the ventromedian cells are more substantial (Figure 2A,D,F). The cytoplasm of ventromedian cells is homogenous, finely granulated and stains weakly to medium violet with toluidine blue in light micrographs (Figures 2, 4, 5).
2. **Gland cells** make up most of the endostyle's volume. They are stained in dark violet with toluidine blue in light micrographs and are filled with extensive arrays of cisternae of rough endoplasmic reticulum (Figures 2, 3, 4). Gland cells are large, measuring at least 30 *µm (e.g. Fritillaria borealis*, Figure 2H) and up to more than 100 µm (*Bathochordaeus stygius*, Figures 2B, 3F,G) in diameter. They rest basally on a basement membrane and possess vesicles that are more concentrated apically. Apical cilia are present. In *F. borealis*, numerous microvilli are interspersed among the apical cilia (Figure 4C‐E).

The number of gland cells within the endostyle varies among species. Species within the genus *Oikopleura* exhibit a relatively consistent gland cell count, with *Oikopleura dioica* possessing 38 cells and *Oikopleura vanhoeffeni* 40. Larger species like *Bathochordaeus stygius* possess 42 gland cells, while *Megalocercus huxleyi* features more cells (74) and *Stegosoma magnum* has 80. *Folia mediterranea* shows an intermediate count of 46 cells. In the genus *Fritillaria*, endostyles are characterized by lower numbers, with *Fritillaria pellucida* having 18 gland cells and *Fritillaria borealis* 24.

Similarly, the arrangement of glandular cells in the endostyle of appendicularians varies among species. In *Megalocercus huxleyi, Bathochordaeus stygius*, and *Stegosoma magnum*, two rows of glandular cells on each side can be found (Figures 2, 3, 5). In *B. stygius* and *S. magnum* the gland cells in the ventral rows are smaller in cross sections. Two rows of gland cells are present in *Oikopleura vanhoeffeni*, but the ventral row is shortened considerably compared to the dorsal row; the ventral row of gland cells is present only in the anterior portion of the endostyle. In contrast, *Oikopleura dioica, Folia mediterranea, Fritillaria pellucida*, and *Fritillaria borealis* all possess only a single row of glandular cells on each side (Figures 2E–H, 4, 5).

1. **Ciliated cells** form a row of cells along the anteroposterior axis of the endostyle between the ventral and dorsal row of gland cells in *B. stygius, S. magnum* and *M. huxleyi*. In *O. dioica, O. vanhoeffeni* and *F. mediterranea* the ciliated cells form rows of cells that run parallel to the ventromedian cells on either side. In fritillariids, the ciliated cells are restricted to the ventral midline of the endostyle (Figure 2). The ciliated cells are typical epithelial cells that rest on a basal membrane and possess apical cilia. In cross sections, ciliated cells are either cuboidal or with a narrow basal region and an expanding, bulging apical area. Numerous cilia are present on the apical surfaces of ciliated cells. The cytopolasm stains faintly in touidine blue‐stained light micrographs (Figures 2, 3). Transmission electron micrographs in *F. borealis* show no microvilli between the apical cilia (Figure 4E).
2. **Connecting cells** are small cells, approximately cuboidal in cross sections. They lie between the corridor cells, which connect to the pharynx epithelium dorsally, and the gland cells ventrally (Figures 2, 5). Connecting cells are distinguished by their lighter appearance in toluidine blue‐stained light micrographs.
3. **Corridor cells** in cross sections, a short row of cells on each side connects the endostyle with the epithelium of the pharynx, forming a narrow corridor or channel that opens into the pharynx. The corridor cells are ovoid to cuboid in cross sections, and possess a central ovoid nucleus. Their cytoplasm stains lightly to moderately bluish‐violet in toluidine blue‐stained light micrographs (Figures 2, 3).
4. **Bright cells** are the most anterior pair of cells in the dorsal row of gland cells, found in some appendicularians immediately next to the giant cilia cells. They resemble gland cells but are easily distinguished by their conspicuously lighter coloration with a hue to the red in toluidine blue‐stained light micrographs. Bright cells possess regularly arranged cilia along their apical surface (Figures 2A, 3A,C,D,G). In transmission electron micrographs of *F. borealis*, bright cells are less electron‐dense than the adjacent gland cells. Like gland cells, bright cells contain a large amount of rough endoplasmic reticulum (Figure 4), that is, however, less densely packed than in the gland cells and with more dilated cisternae. Bright cells were found in four of the six oikopleurid species analyzed (*M. huxleyi, B. stygius, S. magnum, O. vanhoeffeni*) and in one of four fritillariid species (*F. borealis*). In the endostyles of *M. huxleyi* and *S. magnum* with a generally larger number of gland cells, there are two pairs of bright cells on each side, while in the remaining species a single pair is present. In other species, all cells in the dorsal row of gland cells look alike.
5. **Giant cilia cells** are cells with exceptionally long apical cilia which protrude into the endostyle lumen (Figures 2B, 3). The giant cilia cells are located anteriorly between the lateral rows of gland cells. Four to six giant cilia cells are seen arranged in a row on cross sections (Figures 3, S1). The cilia of the giant cilia cells are the longest cilia found in the endostyle with some reaching from the endostyle through the opening into the pharynx (Figures 2A,D,E,F, S1A). Giant cilia cells are present in oikopleurids but absent in fritillariids (Figures 2, 3).

### 3.3 Ciliary Bands

**Figure 6:**
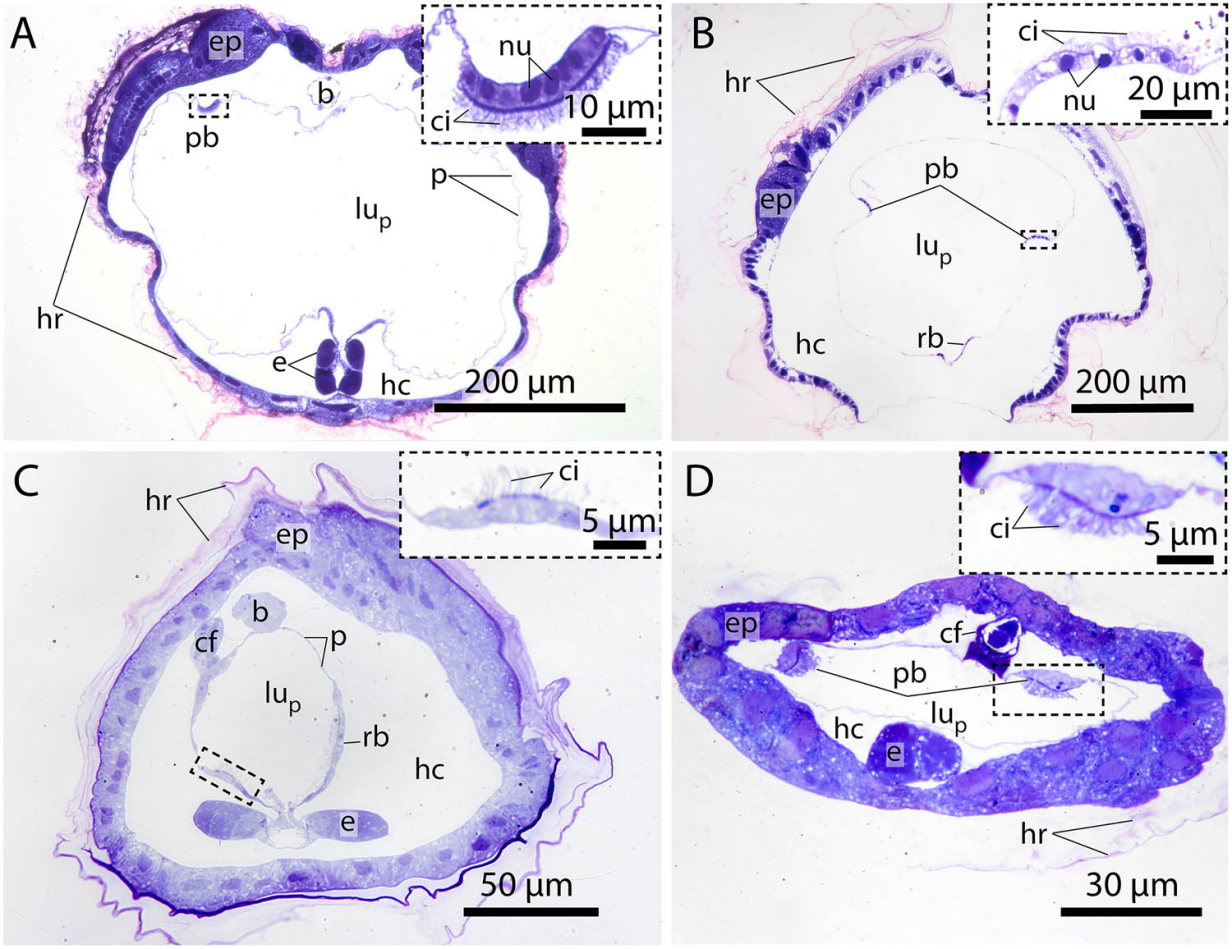
Light micrographs of cross sections through the pharynx of different appendicularian species to show ciliated bands. Dashed rectangles indicate areas enlarged in insets (upper right). (A) *Megalocercus huxleyi*, (B) *Oikopleura vanhoeffeni*. (C) *Oikopleura dioica*. (D) *Fritillaria borealis*. b – brain, e – endostyle, cf – ciliated funnel, ci – cilia, ep – epidermis, hc – hemocoel, hr – house rudiment, lu_p_ – lumen of pharynx, nu – nucleus, pb – peripharyngeal band, rb – retropharyngeal band.

**Figure 7:**
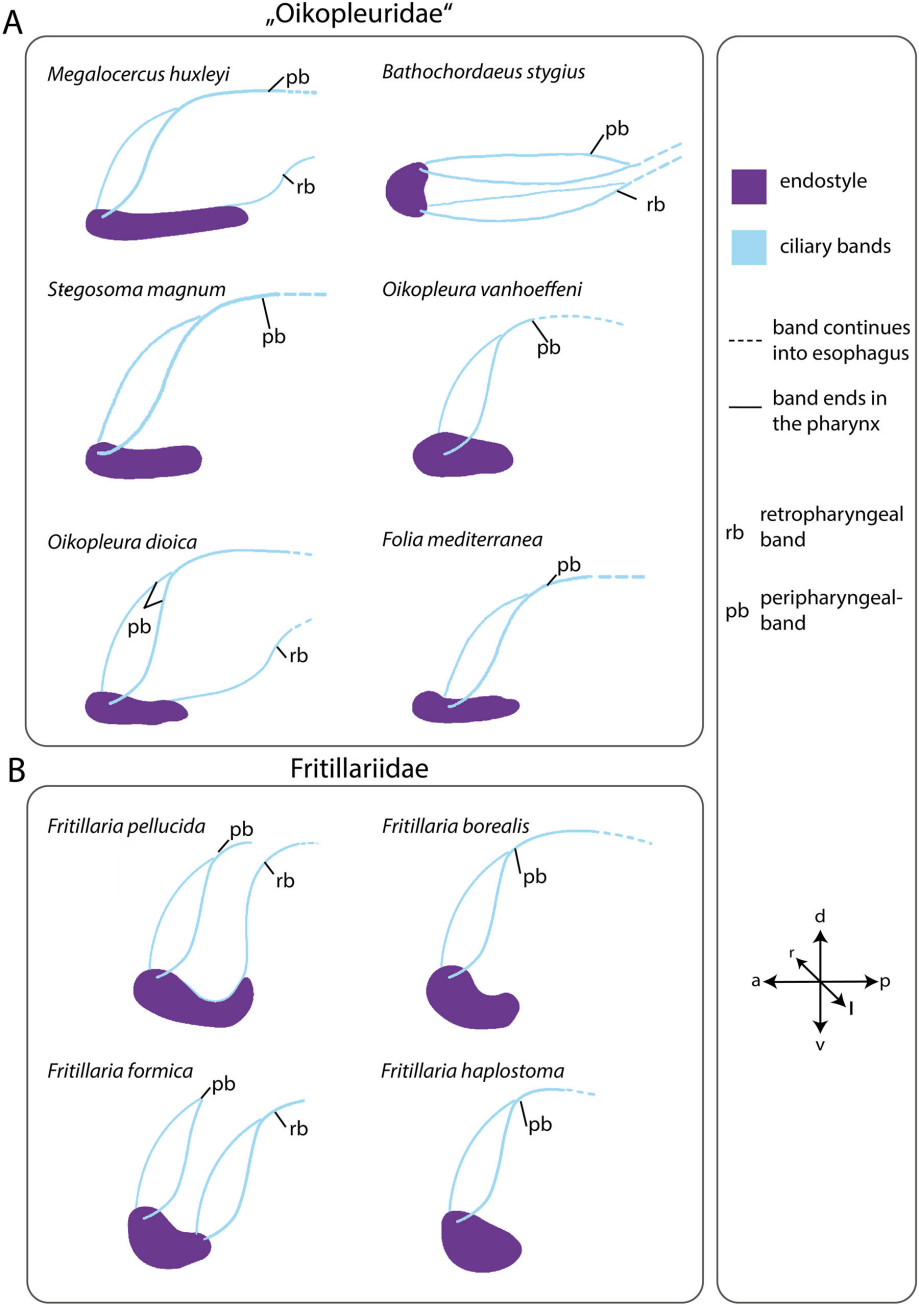
Illustrations of ciliary bands and their relation to the endostyle in 12 appendicularian species. (A, B) Dotted lines indicate the continuation of ciliary bands into the esophagus.

Ciliary bands running alongside the pharyngeal walls originate at the endostyle and continue into the esophagus (Figure 6). In many species, two pairs of ciliary bands are present: the peripharyngeal band originates at the anterior end of the endostyle and a retropharyngeal band originates at the posterior end of the endostyle (Figure 7).

The peripharyngeal band is present in all species with an endostyle. It runs obliquely posterior on either side of the pharynx and the two bands fuse at the dorsal side. The fused peripharyngeal band runs into the esophagus in all species except for *Fritillaria pellucida* and *F. formica*. In the latter two species, the peripharyngeal band ends just at the entrance to the esophagus.

The retropharyngeal band also runs obliquely to the posterior in *F. formica*, merges, and enters the esophagus. The retropharyngeal bands extend as a single band from the posterior of the endostyle in *M. huxleyi*, *O. dioica*, and *F. pellucida*. Retropharyngeal bands are missing in *S. magnum*, *Folia mediterranea*, *Fritillaria borealis*, and *F. haplostoma*.

In *B. stygius* the peripharyngeal band and the retropharyngeal band run parallel to each other horizontally (Figure 7).

## 4 Discussion

In the present study, we examined the pharyngeal complexes of twelve appendicularian species across the three classically recognized families: Oikopleuridae, Fritillariidae and Kowalevskiidae. Our detailed 3D‐reconstructions of endostyles and ciliary bands document a diversity that is phylogenetically informative and functionally significant.

Ihle (1907) and Lohmann (1933) already recognized that appendicularian endostyles were not uniform, and the latter distinguished between a more rod‐like shape in Oikopleuridae and a more compact and curved form in Fritillariidae. The third family, Kowalevskiidae, lacks endostyles, and we could not detect any glandular vestiges in complete serial sections. Our analysis confirmed these previous findings in general, added substantial details, and resulted in a different phylogenetic interpretation.

**Figure 8:**
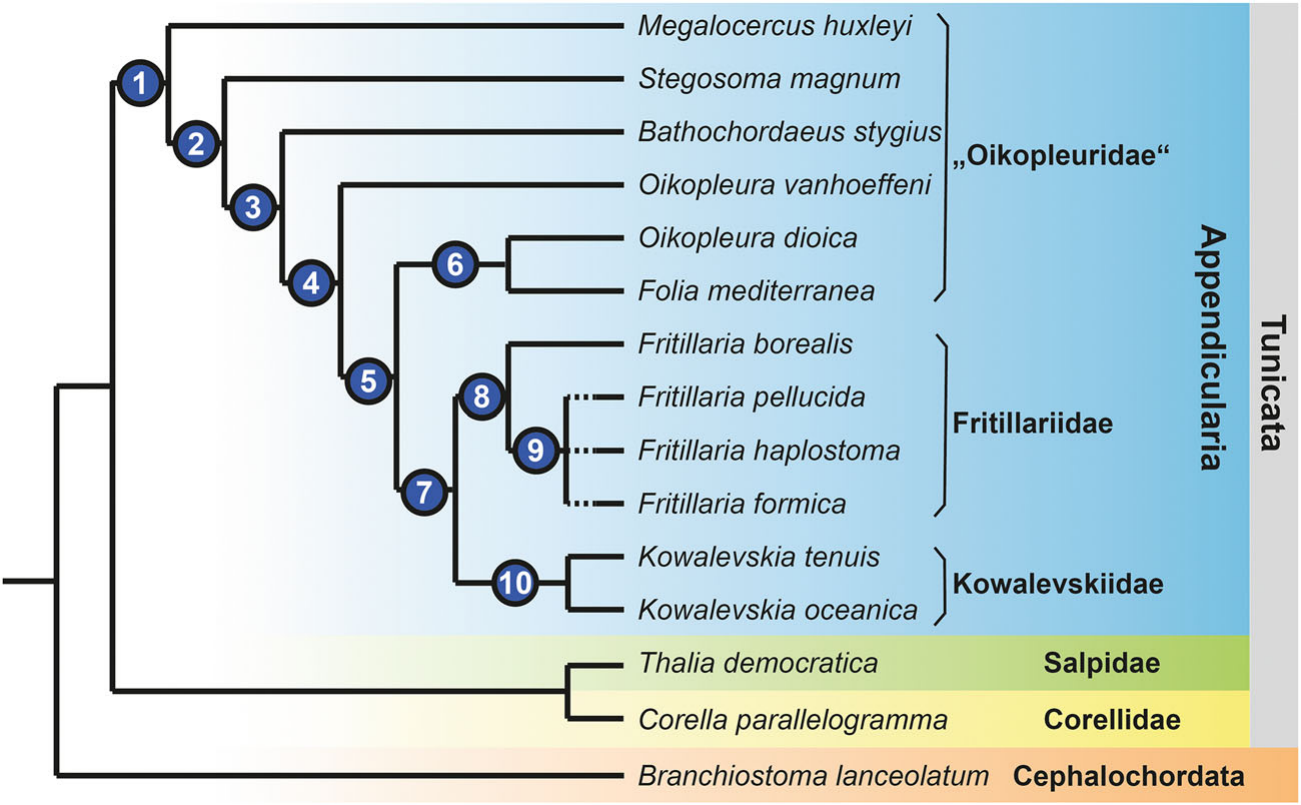
Phylogenetic hypothesis, based on the distribution of endostyle and ciliary band characters. Apomorphic changes are inferred for the respective nodes (see text for details): 1: Reduction of the number of gland cells in gland cell rows, reduction of giant cilia cells, and concentration towards the anterior end, shortening of the endostyle. 2: Partial reduction in size of the ventral gland cell row, further shortening of the endostyle. 3: Reduction of gland cells from the posterior end of the endostyle, further shortening of the endostyle, fewer than 50 gland cells. 4: Almost complete loss of the ventral gland cell row. 5: Complete loss of the ventral gland cell row. 6: Loss of bright cells (homoplastic in 9), endostyle shape broad and dorsoventrally flattened. 7: Loss of ventromedian cells, loss of giant cilia cells, fewer than 25 gland cells. 8: Dorsoventral curvature (sensu Lohmann 1933). 9: Loss of bright cells (homoplastic to 6). 10: Complete loss of the endostyle and adaptations to the morphology of the pharyngeal complex.

Using cladistic argumentation (e.g., Hennig 1950; Wiley and Lieberman 2011), outgroup comparison shows that a straight, rod‐like endostyle is present in other tunicates, i.e., ascidians (e.g., Holley 1986; Burighel and Cloney 1997) and thaliaceans (e.g., Compère and Godeaux 1997; Bone et al. 2000), but also in cephalochordates (Fredriksson et al. 1984; Ruppert 1997a). A rod‐shaped endostyle is therefore plesiomorphic in Appendicularia and – contra Lohmann (1933) – cannot be used to support the monophyly of Oikopleuridae. The apomorphic state – a compact and curved endostyle – on the other hand, could either be interpreted to support Fritillariidae or a sistergroup relationship between Fritillariidae and Kowalevskiidae. In the latter case, a further, complete reduction would be apomorphic for the monophyletic Kowalevskiidae (Figure 8). While these different hypotheses cannot be resolved based on the currently available data, a more detailed comparative phylogenetic analysis of endostyles is possible. Again, cladistic outgroup comparisons allow for a more detailed resolution of endostyle shapes as a phylogenetically informative character and to also assess the cellular composition of endostyles phylogenetically.

A major component of the endostyle is rows of glandular cells. In cephalochordates (Olsson 1963; Fredriksson et al. 1984; Ruppert 1997a) and thaliaceans (Compère and Godeaux 1997; Bone et al. 2000) there are two longitudinal rows of numerous large gland cells. In appendicularians we found species with two rows of single gland cells and species that showed only one row of single gland cells. This character distribution supports an apomorphic reduction from two rows consisting of files of numerous gland cells to two rows consisting of gland cells in single file in the stem lineage of Appendicularia. Moreover, this character distribution indicates that a reduction from two rows of glandular cells to a single row is apomorphic within Appendicularia. Because some species considered oikopleurids (*O. dioica*, *O. vanhoeffeni*, *Folia mediterranea*) together with all fritillariid species possess also a single row of gland cells in their endostyles, these species can be inferred to be more closely related to fritillariids than to other oikopleurid species. In other words, we propose that ‘Oikopleuridae’ is paraphyletic. What about Kowalevskiidae? In the two species of kowalevskiids, endostyles are completely reduced, and this condition can be understood as a further reduction from the single row of gland cells. The sister taxon to Kowalevskiidae must therefore possess a single row of gland cells.

Considering the plesiomorphic state with two rows of gland cells, the question arises, which of the rows was reduced during evolution? Here, the morphology of endostyles in *Megalocercus huxleyi*, *Stegosoma magnum*, *Bathochordaeus stygius*, and *Oikopleura vanhoeffeni* becomes interesting. These species can be arranged in a series, where the ventral row of gland cells is consecutively reduced, each of the species representing a state of apomorphic reduction: *M. huxleyi* with two rows of almost identical sizes, *S. magnum* with a still elongated, yet already shortened, and smaller‐sized ventral row, *B. stygius* with shortened dorsal and ventral rows, and *O. vanhoeffeni* with a barely recognizable remnant of the ventral row of gland cells. Incidentally, the overall shape of endostyles concurs with this hypothesis of a stepwise reduction: *M. huxleyi* shows an elongated (length: width ratio [lwr]: 4.7), rod‐like endostyle that is still similar to the endostyle shapes observed in cephalochordates and other (non‐appendicularian) tunicates, *S. magnum* (lwr: 2.6), and the remaining ‘oikopleurids’ a clearly shorter endostyle (lwr: < 2.0). A similar scenario of reduction has been proposed by Ihle (1907, 1908), who adds *O. rufescens* to the species with two rows of gland cells; however, at the same time admits that the quality of the material examined leaves room for doubt.

Based on transmission electron micrographs, Olsson (1965) described six different cell types in the endostyle of *O. dioica*. From ventral to dorsal, these cell types are: ventromedian cells, gland cells, ciliated cells, connecting cells, and corridor cells. Giant cilia cells, the sixth type, are situated anteriorly, dorsally in the midline of the endostyle. Adjacent to these giant cilia cells, we found a seventh cell type in four of the six oikopleurid species (*M. huxleyi*, *S. magnum*, *B. stygius*, *O. vanhoeffeni*). Based on its histological appearance, we named this cell type ‘bright cells’. A pair of bright cells is also present in *Fritillaria borealis* in a similar position as in the oikopleurid species, while in other fritillariids, no bright cells were present. All fritillariids examined, including *F. borealis*, lack giant cilia cells. The character distribution of the presence of bright cells in Appendicularia suggests that bright cells were present in the last common ancestor of Appendicularia and were lost probably three times independently: once within oikopleurid species, once within Fritillariidae, and another time in the stem lineage of Kowalevskiidae.

Bright cells have not been reported from non‐appendicularian tunicates nor from cephalochordates (Olsson 1963; Burighel and Cloney 1997; Ruppert 1997a, Bone et al. 2000). However, the most anterior ends of endostyles, where we would expect bright cells to be located, have not been studied in detail in these taxa. On the other hand, bright cells have also not been described in *M. huxleyi*, the only species where we found bright cells that had been examined in a classical light‐microscopical study (Ihle 1907). Perhaps our improved microscopic approach with resin‐embedding serial semi‐thin sectioning and subsequent 3D‐reconstruction is the reason we were able to detect these cells. Bright cells are not an artifact of preparation, as we found them consistently in the same position, independent of the fixation of the specimens. Moreover, we could reproduce the lack of bright cells in *O. dioica* (Olsson 1965). In toluidine‐blue stained sections, bright cells resemble gland cells, but are lighter stained with a reddish hue, indicating a slightly decreased pH (Weissman et al. 1952; Bergholt et al. 2019). Transmission electron micrographs in *F. borealis* reveal a less electron‐dense cytoplasm and expansive rough endoplasmic reticulum with larger lumina and likewise less electron‐dense content compared to the gland cells (Figure 4A,B). The bright cells are therefore glandular yet clearly distinguishable from the neighboring gland cells; both cell types appear to produce different substances, possibly reflecting the different mechanical needs of the mucus net at the anterior rim.

In addition to the endostyle per se, we also compared the ciliated bands associated with the endostyles. An anterior peripharyngeal and a posterior retropharyngeal band are present in ascidians and thaliaceans (e.g., Huus 1956; Braun and Stach 2016) and cephalochordates (Franz 1927; Ruppert 1997a). In appendicularians, two ciliated bands, a peripharyngeal and a retropharyngeal band are present in oikopleurid and fritillariid species. In several fritillariid and oikopleurid species only the peripharyngeal band is present, indicating that the retropharyngeal band is evolutionarily lost independently several times.

Based on considerations of endostyle and pharyngeal characters, we can suggest an evolutionary hypothesis where consecutive and repeated reductions from an elongated rod‐like ventral gland with two rows of glandular cells through shorter and curved endostyles to a complete reduction of endostyles in kowalevskiids (Figure 8). Endostyle and pharyngeal characters indicate that ‘Oikopleuridae’ might be paraphyletic.

Morphological disparity is not limited to the endostyle. Especially the disparity of the intestinal tract has been used in taxonomic classification (e.g., Lohmann 1933; Tokioka 1955; Fenaux 1998b), and detailed ultrastructural studies of the intestinal tracts exist for *O. dioica* (Burighel et al. 2001), *O. gracilis* (Savelieva 2023), *Kowalevskia tenuis* and *K. gracilis* (Brena et al. 2003a), and *Fritillaria formica* and *F. pellucida* (Brena et al. 2003b). Lohmann (1933) summarized and appended classical light‐microscopical information on other morphologically diverse structures in appendicularians, such as the pattern of oikoplast cells, or subchordal cells. Holland et al. (2005) investigated the ultrastructure of spermatozoa in appendicularian species from all three classically recognized families and documented considerable variation in several species across the major appendicularian taxa. Their analysis revealed striking differences in head morphology, acrosomal configuration, and flagellar architecture, culminating in the question of whether Appendicularia constitutes a monophyletic group. In some cases, sperm traits in one appendicularian family were more similar to those found in other tunicate ‘classes’ than to those of other appendicularian ‘families’. Whereas these studies document the high morphological disparity, potentially reflecting deep evolutionary splits within Appendicularia, no study has used cladistic methodology to infer a phylogenetic hypothesis based on morphological characters. Nevertheless, this morphological heterogeneity across independently evolving organ systems further strengthens the case for reconsidering traditional classifications.

Focusing on the morphology of endostyles, we document their histological composition, suggest hypotheses of homology, and present a first phylogenetic hypothesis based on the conceptualization of endostyle‐related characters. The evolutionary trends of reductions inferred from this phylogenetic hypothesis can be seen in a functional context. A hallmark of appendicularian biology is the evolution of an external complex filtering device, the so‐called house (e.g., Lohmann 1933; Flood 2003; Razghandi et al. 2021). This house, at the same time, increases the concentration of food that enters the mouth and reduces the necessity for numerous gill openings. The internal filtering mucus net that is crucial in ascidians (e.g., Flood and Fiala‐Medioni 1981; Riisgård and Larsen 2010) and cephalochordates (Nash 2002; Nielsen et al. 2007), likely plays a decreasing role in appendicularians. Because food particles still need to be retained and prevented from leaving the pharynx through the gill openings, the endostyle is retained in most appendicularian species, albeit in a reduced state. In fritillariids, where the opening of the endostyle groove to the pharynx is reduced in size and giant cilia cells are missing, the house functions in a distinctly different way and is inflatable and reusable (Flood 2003). In fact, in kowalevskiids, where the endostyle is lost without a trace, the comb‐like projections dorsal and ventral in the pharynx retain ingested particles with their cilia. Unfortunately, we know little about the differences in ecological requirements, such as natural food composition or predator interactions, in the different appendicularian species and therefore we have only a limited understanding of how ecological necessities result in the evolution of the different shapes of houses and endostyles.

Another function of endostyles in non‐appendicularian species is their role in regulating metamorphosis through the production of iodinated thyroxine hormones. This has been shown in ascidians (Cloney 1982; Sasakura and Hozumi 2018), cephalochordates (Barrington 1962; Paris and Laudet 2008; Paris et al. 2010), and lampreys (Youson 1997; Manzon and Manzon 2017). Fredriksson et al. (1985, 1989) have shown that corridor cells in the endostyles of *O. dioica*, *O. albicans*, and *O. longicauda* bind iodine. Metamorphosis in appendicularians is little known and considered to consist mainly of the shift of the tail from the larval posterior position to the ventral side of the trunk (Delsman. 1912; Stach et al. 2008; Nishida et al. 2021).

## 5 Conclusion

In summary, we documented a considerable disparity of endostyle anatomies within Appendicularia, identified a formerly undescribed type of cells, the bright cells, and analyzed endostyle characters in a cladistic paradigm. This analysis resulted in a phylogenetic hypothesis that suggests that “Oikopleuridae” is a paraphyletic grouping and supports an evolutionary scenario with multiple reductions that could be functionally related to the evolution of the external filter house of appendicularians. A more detailed cladistic analysis, including other organ systems, is needed to resolve the phylogenetic relationships and to understand the evolution of appendicularian taxa.

## Author Contributions

**Mai‐Lee Van Le:** conceptualization, investigation, writing – original draft, methodology, validation, visualization, writing – review and editing, software, formal analysis, data curation, supervision, resources. **Seowon Park:** investigation, writing – review and editing, formal analysis, data curation. **Thomas Stach:** conceptualization, funding acquisition, writing – original draft, validation, writing – review and editing, supervision, project administration, resources.

## Peer Review

The peer review history for this article is available at https://www.webofscience.com/api/gateway/wos/peer-review/10.1002/jmor.70061.

## Supporting information

Endostyle manuscript 2025 02 13 rev 2025 06 06 changes accepted supplementary material.

## Data Availability

The data that support the findings of this study are available on request from the corresponding author. We would like to deposit movie files of the aligned microscopic images as supporting information.

## References

[jmor70061-bib-0001] Alldredge, A. L. , G. Gorsky , M. Youngbluth , and D. Deibel . 2005. “The Contribution of Discarded Appendicularian Houses to the Flux of Particulate Organic Carbon From Oceanic Surface Waters.” In Response of Marine Ecosystems to Global Change: Ecological Impact of Appendicularians, edited by G. Gorsky , M. Youngbluth , and D. Deibel , 315–332. Contemporary Publishing International.

[jmor70061-bib-0002] Barrington, E. J. W. 1962. “Hormones and Vertebrate Evolution.” Experientia 18, no. 5: 201–209. 13865266 10.1007/BF02148301

[jmor70061-bib-0003] Bergholt, N. L. , H. Lysdahl , M. Lind , and C. B. Foldager . 2019. “A Standardized Method of Applying Toluidine Blue Metachromatic Staining for Assessment of Chondrogenesis.” Cartilage 10, no. 3: 370–374. 29582671 10.1177/1947603518764262 PMC6585293

[jmor70061-bib-0004] Bone, Q. , C. Carre , and K. P. Ryan . 2000. “The Endostyle and the Feeding Filter in Salps (Tunicata).” Journal of the Marine Biological Association of the United Kingdom 80, no. 3: 523–534.

[jmor70061-bib-0005] Braun, K. , and T. Stach . 2016. “Comparative Study of Serotonin‐Like Immunoreactivity in the Branchial Basket, Digestive Tract, and Nervous System in Tunicates.” Zoomorphology 135: 351–366.

[jmor70061-bib-0006] Brena, C. , F. Cima , and P. Burighel . 2003a. “Alimentary Tract of Kowalevskiidae (Appendicularia, Tunicata) and Evolutionary Implications.” Journal of Morphology 258, no. 2: 225–238. 14518015 10.1002/jmor.10145

[jmor70061-bib-0007] Brena, C. , F. Cima , and P. Burighel . 2003b. “The Highly Specialised Gut of Fritillariidae (Appendicularia: Tunicata).” Marine Biology 143: 57–71.

[jmor70061-bib-0008] Bucklin, A. , K. T. C. A. Peijnenburg , K. N. Kosobokova , et al. 2021. “Toward a Global Reference Database of COI Barcodes for Marine Zooplankton.” Marine Biology 168, no. 6: 78.

[jmor70061-bib-0009] Burighel, P. , C. Brena , G. B. Martinucci , and F. Cima . 2001. “Gut Ultrastructure of the Appendicularian *Oikopleura dioica* (Tunicata).” Invertebrate Biology 120, no. 3: 278–293.

[jmor70061-bib-0010] Burighel, P. , and R. A. Cloney . 1997. “Urochordata: Ascidiacea.” In Microscopic Anatomy of Invertebrates, edited by F. W. Harrison and E. E. Ruppert , 15, 221–347. Willey‐Liss, Incorporation.

[jmor70061-bib-0011] Cañestro, C. , S. Bassham , and J. H. Postlethwait . 2008. “Evolution of the Thyroid: Anterior–Posterior Regionalization of the *Oikopleura* Endostyle Revealed by Otx, Pax2/5/8, and Hox1 Expression.” Developmental Dynamics 237, no. 5: 1490–1499. 18386819 10.1002/dvdy.21525

[jmor70061-bib-0012] Cloney, R. A. 1982. “Ascidian Larvae and the Events of Metamorphosis.” American Zoologist 22, no. 4: 817–826.

[jmor70061-bib-0013] Compère, P. , and J. E. A. Godeaux . 1997. “on Endostyle Ultrastructure in Two New Species of Doliolid‐Like Tunicates.” Marine Biology 128: 447–453.

[jmor70061-bib-0014] Conley, K. R. , B. J. Gemmell , J. M. Bouquet , E. M. Thompson , and K. R. Sutherland . 2018. “A Self‐Cleaning Biological Filter: How Appendicularians Mechanically Control Particle Adhesion and Removal.” Limnology and Oceanography 63, no. 2: 927–938.

[jmor70061-bib-0015] Deibel, D. , P. A. Saunders , J. L. Acuna , A. B. Bochdansky , N. Shiga , and R. B. Rivkin . 2005. “The Role of Appendicularian Tunicates in the Biogenic Carbon Cycle of Three Arctic Polynyas.” In Response of Marine Ecosystems to Global Change: Ecological Impact of Appendicularians, edited by G. Gorsky , M. Youngbluth , and D. Deibel , 327–358. Contemporary Publishing International.

[jmor70061-bib-0016] Delsman, H. C. 1912. “Weitere Beobachtungen Über Die Entwicklung Von *Oikopleura dioica* .” Tijdschr Ned Dierk Ver (Ser 2) 12: 197–215.

[jmor70061-bib-0017] Fenaux, R. 1998a. “Anatomy and Functional Morphology of the Appendicularia.” In The Biology of Pelagic Tunicates, edited by Q. Bone , 25–34. Oxford University Press.

[jmor70061-bib-0018] Fenaux, R. 1998b. “The Classification of Appendicularia.” In The Biology of Pelagic Tunicates, edited by Q. Bone , 295–306. Oxford University Press.

[jmor70061-bib-0019] Ferrández‐Roldán, A. , J. Martí‐Solans , C. Cañestro , and R. Albalat . 2019. “ *Oikopleura dioica*: An Emergent Chordate Model to Study the Impact of Gene Loss on the Evolution of the Mechanisms of Development.” In Evo‐devo: Non‐model Species in Cell and Developmental Biology, 63–105. 10.1007/978-3-030-23459-1_4 31598853

[jmor70061-bib-0021] Flood, P. R. 1994. “Appendicularian Houses–Architectural Wonders of the Sea.” In *Evolution of Natural Structures*. Proceedings of the 3rd International Symposium Sonderforschungsbereich (Vol. 230).

[jmor70061-bib-0022] Flood, P. R. 2003. ““House Formation and Feeding Behaviour of *Fritillaria borealis* (Appendicularia: Tunicata).” Marine Biology 143, no. 3: 467–475.

[jmor70061-bib-0023] Flood, P. R. , and A. Fiala‐Medioni . 1981. “Ultrastructure and Histochemistry of the Food Trapping Mucous Film in Benthic Filter‐Feeders (Ascidians).” Acta Zoologica 62, no. 1: 53–65.

[jmor70061-bib-0024] Franz, V. 1927. “Morphologie Der Akranier.” Zeitschrift für die gesamte Anatomie: Abt. 3 27, no. 27: 464–692.

[jmor70061-bib-0025] Fredriksson, G. , L. E. Ericson , and R. Olsson . 1984. “Iodine Binding in the Endostyle of Larval *Branchiostoma lanceolatum* (Cephalochordata).” General and Comparative Endocrinology 56: 177–184. 6510680 10.1016/0016-6480(84)90028-5

[jmor70061-bib-0026] Fredriksson, G. , R. Fenaux , and L. E. Ericson . 1989. “Distribution of Peroxidase and Iodination Activity in the Endostyles of *Oikopleura albicans* and *Oikopleura longicauda* (Appendicularia, Chordata).” Cell and Tissue Research 255: 505–510.

[jmor70061-bib-0027] Fredriksson, G. , T. Öfverholm , and L. E. Ericson . 1985. “Ultrastructural Demonstration of Iodine Binding and Peroxidase Activity in the Endostyle of *Oikopleura Dioica* (Appendicularia).” General and Comparative Endocrinology 58, no. 2: 319–327. 2987082 10.1016/0016-6480(85)90348-x

[jmor70061-bib-0028] Fredriksson, G. , T. Öfverholm , and L. Ericson . 1988. “Iodine Binding and Peroxidase Activity in the Endostyle of *Salpa fusiformis*, *Thalia democratica*, *Dolioletta gegenbauri* and *Doliolum nationalis* (Tunicata, Thaliacea).” Cell and Tissue Research 253: 403–411. 3409292 10.1007/BF00222297

[jmor70061-bib-0029] Grau‐Bové, X. , L. Subirana , L. Meister , et al. 2024. “An Amphioxus Neurula Stage Cell Atlas Supports a Complex Scenario for the Emergence of Vertebrate Head Mesoderm.” Nature Communications 15, no. 1: 4550. 10.1038/s41467-024-48774-4 PMC11136973 38811547

[jmor70061-bib-0030] Hennig, W. 1950. “Grundzüge einer Theorie der phylogenetischen Systematik.” *Deutscher Zentralverlag*.

[jmor70061-bib-0031] Hiruta, J. , F. Mazet , K. Yasui , P. Zhang , and M. Ogasawara . 2005. “Comparative Expression Analysis of Transcription Factor Genes in the Endostyle of Invertebrate Chordates.” Developmental Dynamics 233, no. 3: 1031–1037. 15861404 10.1002/dvdy.20401

[jmor70061-bib-0032] Holland, L. Z. , G. Gorsky , and R. Fenaux . 2005. “A Diversity of Sperm in Appendicularians: Are Appendicularians Monophyletic?” In Response of Marine Ecosystems to Global Change: Ecological Impact of Appendicularians, edited by G. Gorsky , M. Youngbluth , and D. Deibel , 27–41. Contemporary Publishing International.

[jmor70061-bib-0033] Holland, L. Z. , V. Laudet , and M. Schubert . 2004. “The Chordate Amphioxus: An Emerging Model Organism for Developmental Biology.” Cellular and Molecular Life Sciences 61: 2290–2308. 15378201 10.1007/s00018-004-4075-2 PMC11138525

[jmor70061-bib-0034] Holley, M. C. 1986. “Cell Shape, Spatial Patterns of Cilia, and Mucus‐Net Construction in the Ascidian Endostyle.” Tissue and Cell 18, no. 5: 667–684. 18620176 10.1016/0040-8166(86)90069-8

[jmor70061-bib-0035] Hopcroft, R. R. 2005. “Diversity in Larvaceans: How Many Species?” In Response of Marine Ecosystems to Global Change: Ecological Impact of Appendicularians, edited by G. Gorsky , M. Youngbluth , and D. Deibel , 45–57. Contemporary Publishing International.

[jmor70061-bib-0036] Huus, J. 1956. “Zweite und letzte Unterklasse der Acopa: Ascdiacea = Tethyodeae = Seescheiden.” In *Handbuch* d*er Zoologie, Fünfter Band, Zweite Hälfte* , edited by T. Krumbach , 545–692. Walter de Gruyter.

[jmor70061-bib-0037] Ihle, J. E. W. 1907. “ber Den Endostyl Und Die Systematische Stellung Der Appendicularien.” Zoologischer Anzeiger 31: 770.

[jmor70061-bib-0038] Ihle, J. E. W. 1908. *Die Appendicularien der* S*iboga‐Expedition* . E. J. Brill.

[jmor70061-bib-0039] Jiang, A. , K. Han , J. Wei , et al. 2024. “Spatially Resolved Single‐Cell Atlas of Ascidian Endostyle Provides Insight Into the Origin of Vertebrate Pharyngeal Organs.” Science Advances 10, no. 13: eadi9035. 38552007 10.1126/sciadv.adi9035 PMC10980280

[jmor70061-bib-0040] Jiang, A. , W. Zhang , J. Wei , P. Liu , and B. Dong . 2023. “Transcriptional Analysis of the Endostyle Reveals Pharyngeal Organ Functions in Ascidian.” Biology 12, no. 2: 245. 36829522 10.3390/biology12020245 PMC9953650

[jmor70061-bib-0041] Katija, K. , C. A. Choy , R. E. Sherlock , A. D. Sherman , and B. H. Robison . 2017. “From the Surface to the Seafloor: How Giant Larvaceans Transport Microplastics Into the Deep Sea.” Science Advances 3, no. 8: e1700715. 28835922 10.1126/sciadv.1700715 PMC5559207

[jmor70061-bib-0042] Kieckebusch, H. H. 1928. “Beiträge Zur Kenntnis Des Baues Und Der Entwicklung Der Schilddrüse Bei Den Neunaugenlarven (*Lampetra fluviatilis* L. Und *Lampetra planeri* Bl.).” Zeitschrift für Morphologie und Ökologie der Tiere 11, no. 3/4: 247–360.

[jmor70061-bib-0043] Körner, W. F. 1952. “Untersuchungen Über Die Gehäusebildung BEI Appendicularien (*Oikopleura dioica* FOl).” Zeitschrift für Morphologie und Ökologie der Tiere 41: 1–53.

[jmor70061-bib-0044] Lohmann, H. 1933. “Erste Klasse der Tunicaten: Appendiculariae.” In *Handbuch der* Z*oologie, Fünfter Band, Zweite Hälfte* , edited by T. Krumbach , 1–202. Walter de Gruyter.

[jmor70061-bib-0045] Manzon, R. G. , and L. A. Manzon . 2017. “Lamprey Metamorphosis: Thyroid Hormone Signaling in a Basal Vertebrate.” Molecular and Cellular Endocrinology 459: 28–42. 28630022 10.1016/j.mce.2017.06.015

[jmor70061-bib-0046] Müller, W. 1873. ““Über Die Hypobranchialrinne Der Tunicaten Und Deren Vorhandensein Bei Amphioxus Und Den Cyclostomen.” Jena. Z. Med. Naturw 7: 327–332.

[jmor70061-bib-0047] Nash, T. R. 2002. “ *Feeding Biology, Digestive Physiology, and Metabolic Rate of the Florida lancelet*, *Branchiostoma floridae* .” Doctoral Dissertation, Clemson University.

[jmor70061-bib-0048] Nielsen, S. E. , Q. Bone , P. Bond , and G. Harper . 2007. “on Particle Filtration by Amphioxus (*Branchiostoma lanceolatum*).” Journal of the Marine Biological Association of the United Kingdom 87, no. 4: 983–989.

[jmor70061-bib-0049] Nikitina, N. , M. Bronner‐Fraser , and T. Sauka‐Spengler . 2009. “The Sea Lamprey *Petromyzon marinus*: a Model for Evolutionary and Developmental Biology.” Cold Spring Harbor Protocols 2009, no. 1: pdb‐emo113. 10.1101/pdb.emo113 20147008

[jmor70061-bib-0050] Nishida, H. , N. Ohno , F. Caicci , and L. Manni . 2021. “3D Reconstruction of Structures of Hatched Larva and Young Juvenile of the Larvacean *Oikopleura dioica* Using SBF‐SEM.” Scientific Reports 11, no. 1: 4833. 33649401 10.1038/s41598-021-83706-y PMC7921577

[jmor70061-bib-0051] Ogasawara, M. 2000. “Overlapping Expression of Amphioxus Homologs of the Thyroid Transcription factor‐1 Gene and Thyroid Peroxidase Gene in the Endostyle: Insight Into Evolution of the Thyroid Gland.” Development Genes and Evolution 210: 231–242. 11180827 10.1007/s004270050309

[jmor70061-bib-0052] Ogasawara, M. , R. Di Lauro , and N. Satoh . 1999. “Ascidian Homologs of Mammalian Thyroid Transcription factor‐1 Gene Are Expressed in the Endostyle.” Zoological Science 16, no. 3: 559–565. 10.1002/(sici)1097-010x(19990815)285:2<158::aid-jez8>3.0.co;2-0 10440727

[jmor70061-bib-0053] Olsson, R. 1963. “Endostyles and Endostylar Secretions: A Comparative Histochemical Study.” Acta Zoologica 44, no. 3: 299–328.

[jmor70061-bib-0054] Olsson, R. 1965. “The Cytology of the Endostyle of *Oikopleura dioica* .” Annals of the New York Academy of Sciences 118, no. 24: 1038–1051. 5221050 10.1111/j.1749-6632.1965.tb40170.x

[jmor70061-bib-0055] Onuma, T. A. , R. Nakanishi , Y. Sasakura , and M. Ogasawara . 2021. “Nkx2‐1 and Foxe Regionalize Glandular (Mucus‐Producing) and Thyroid‐Equivalent Traits in the Endostyle of the Chordate *Oikopleura dioica* .” Developmental Biology 477: 219–231. 34107272 10.1016/j.ydbio.2021.05.021

[jmor70061-bib-0056] Paris, M. , A. Hillenweck , S. Bertrand , et al. 2010. “Active Metabolism of Thyroid Hormone During Metamorphosis of Amphioxus.” Integrative and Comparative Biology 50, no. 1: 63–74. 21558188 10.1093/icb/icq052

[jmor70061-bib-0057] Paris, M. , and V. Laudet . 2008. “The History of a Developmental Stage: Metamorphosis in Chordates.” Genesis 46, no. 11: 657–672. 18932261 10.1002/dvg.20443

[jmor70061-bib-0058] Razghandi, K. , N. Janßen , M. L. V. Le , and T. Stach . 2021. “The Filter‐House of the Larvacean *Oikopleura dioica*. A Complex Extracellular Architecture: From Fiber Production to Rudimentary State to Inflated House.” Journal of Morphology 282, no. 8: 1259–1273. 34041785 10.1002/jmor.21382

[jmor70061-bib-0059] Richardson, M. K. , J. Admiraal , and G. M. Wright . 2010. “Developmental Anatomy of Lampreys.” Biological Reviews 85, no. 1: 1–33. 19951335 10.1111/j.1469-185X.2009.00092.x

[jmor70061-bib-0060] Riisgård, H. , and P. Larsen . 2010. “Particle Capture Mechanisms in Suspension‐Feeding Invertebrates.” Marine Ecology Progress Series 418: 255–293.

[jmor70061-bib-0061] Robison, B. H. 1993. “Midwater Research Methods With MBARI's ROV.” MTS Journal 26: 32–39.

[jmor70061-bib-0062] Ruppert, E. E. 1997a. “Introduction: Microscopic Anatomy of the Notochord, Heterochrony, and Chordate Evolution.” In Microscopic Anatomy of Invertebrates, edited by F. W. Harrison and E. E. Ruppert , 15, 1–13. Willey‐Liss, Incorporation.

[jmor70061-bib-0063] Ruppert, E. E. 1997b. “Cephalochordata (Acrania).” In Microscopic Anatomy of Invertebrates, edited by F. W. Harrison and E. E. Ruppert , 15, 349–504. Willey‐Liss, Incorporation.

[jmor70061-bib-0064] Salensky, W. 1903. “Etudes Anatomiques Sur Les Appendiculaires. I. *Oikopleura vanhoeffeni* Lohmann.” Mem. Acad. Imp. d. Sciences de St.‐Pétersbourg 13, no. 7: 3–44.

[jmor70061-bib-0065] Salensky, W. 1904. “Etudes Anatomiques Sur Les Appendiculaires. Ii. *Oikopleura rufescens* Fol. – Ii. *Fritillaria pellucida* Busch. – III. *Fritillaria borealis* Lohmann.” Mem. Acad. Imp. d. Sciences de St.‐Pétersbourg 15, no. 1: 1–106.

[jmor70061-bib-0066] Sasakura, Y. , and A. Hozumi . 2018. “Formation of Adult Organs Through Metamorphosis in Ascidians.” WIREs Developmental Biology 7, no. 2: e304. 10.1002/wdev.304 29105358

[jmor70061-bib-0067] Satoh, N. , Y. Satou , B. Davidson , and M. Levine . 2003. “ *Ciona intestinalis*: An Emerging Model for Whole‐genome Analyses.” Trends in Genetics 19, no. 7: 376–381. 12850442 10.1016/S0168-9525(03)00144-6

[jmor70061-bib-0068] Savelieva, A. V. 2023. “Ultrastructural Features of the Alimentary Canal in Hermaphroditic Appendicularians *Oikopleura gracilis* (Tunicata, Oikopleuridae).” Supplement, Russian Journal of Marine Biology 49, no. Suppl 1: S76–S89.

[jmor70061-bib-0069] Stach, T. 2008. “Chordate Phylogeny and Evolution: A Not so Simple Three‐Taxon Problem.” Journal of Zoology 276, no. 2: 117–141.

[jmor70061-bib-0070] Stach, T. , J. Winter , J. M. Bouquet , D. Chourrout , and R. Schnabel . 2008. “Embryology of a Planktonic Tunicate Reveals Traces of Sessility.” Proceedings of the National Academy of Sciences 105, no. 20: 7229–7234. 10.1073/pnas.0710196105 PMC2438232 18490654

[jmor70061-bib-0071] Takagi, W. , F. Sugahara , S. Higuchi , et al. 2022. “Thyroid and Endostyle Development in Cyclostomes Provides New Insights Into the Evolutionary History of Vertebrates.” BMC Biology 20, no. 1: 76. 35361194 10.1186/s12915-022-01282-7 PMC8973611

[jmor70061-bib-0072] Tokioka, T. 1955. “General Consideration on Japanese Appendicularian Fauna.” Publications of the Seto Marine Biological Laboratory 4, no. 2–3: 251–261.

[jmor70061-bib-0073] Weissman, N. , W. H. Carnes , P. S. Rubin , and J. Fisher . 1952. “Metachromasy of Toluidine Blue Induced by Nucleic Acids.” Journal of the American Chemical Society 74, no. 6: 1423–1426.

[jmor70061-bib-0074] Wiley, E. O. , and B. S. Lieberman . 2011. Phylogenetics: Theory and Practice of Phylogenetic Systematics. John Wiley and Sons.

[jmor70061-bib-0075] Wright, G. M. , and J. H. Youson . 1976. “Transformation of the Endostyle of the Anadromous Sea Lamprey, *Petromyzon marinus* L., During Metamorphosis.” General and Comparative Endocrinology 30, no. 3: 243–257. 992347 10.1016/0016-6480(76)90075-7

[jmor70061-bib-0076] Wright, G. M. , and J. H. Youson . 1977. “Serum Thyroxine Concentrations in Larval and Metamorphosing Anadromous Sea Lamprey, *Petromyzon marinus* L.” Journal of Experimental Zoology 202, no. 1: 27–32. 925662 10.1002/jez.1402020104

[jmor70061-bib-0077] Wright, G. M. , and J. H. Youson . 1980. “Transformation of the Endostyle of the Anadromous Sea Lamprey, *Petromyzon marinus* L., During Metamorphosis. Ii. Electron Microscopy.” Journal of Morphology 166, no. 2: 231–257. 30189711 10.1002/jmor.1051660209

[jmor70061-bib-0078] Youson, J. H. 1997. “Is Lamprey Metamorphosis Regulated by Thyroid Hormones?” American Zoologist 37, no. 6: 441–460.

