# Supplementary material for: Comparative 3D‐anatomy of Appendicularian Endostyles (Tunicata, Chordata) ‐ A Tale of Reduction
Source: J Morphol. 2025 Jun 28;286(7):e70061. doi: 10.1002/jmor.70061 (PMC12205476; doi:10.1002/jmor.70061)
Supplement: Supplementary file 1 — Endostyle manuscript 2025 02 13 rev 2025 06 06 changes accepted supplementary material. [file JMOR-286-e70061-s001.docx]

**Supplement**

**
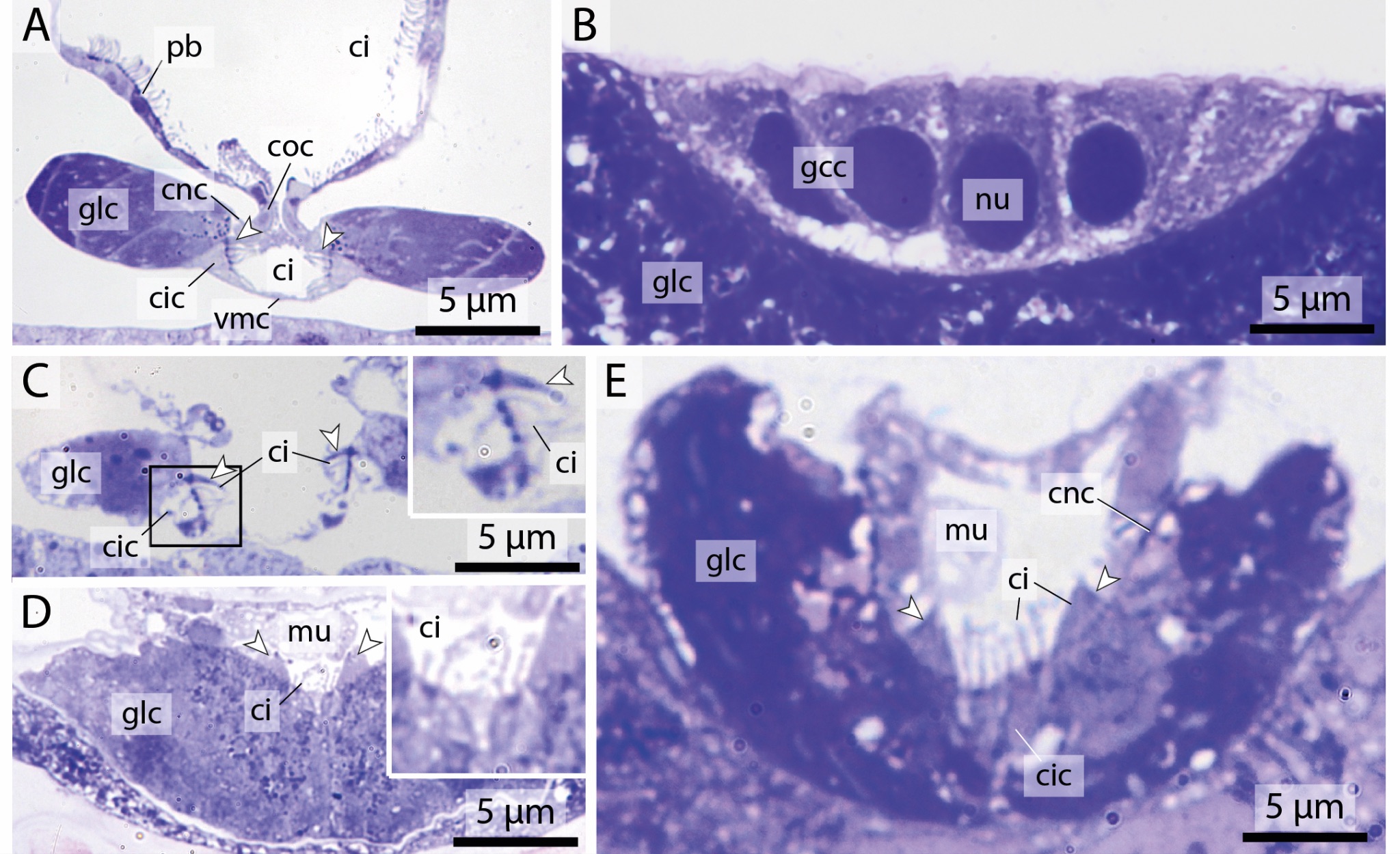
**

**Supplemental Figure S1.** Light micrographs of endostyle cross sections illustrating different cell types. **A:** *Oikopleura dioica*. **B:** *Oikopleura vanhoeffeni*. **C:** *Folia mediterranea*. **D:** *Fritillaria pellucida*. **E:** *Fritillaria borealis*. **bc** – bright cell, **ci** – cilia, **cic** – ciliated cell, **ci_g_** – giant cilia, **cnc** – connecting cell, **coc** – corridor cell, **gcc** – giant cilia cell, **glc** – gland cell, **mu** – mucus, **nu** – nucleus, **pb** – peripharyngeal band, **vmc** – ventromedian cell, arrowheads point to cilia on gland cells.


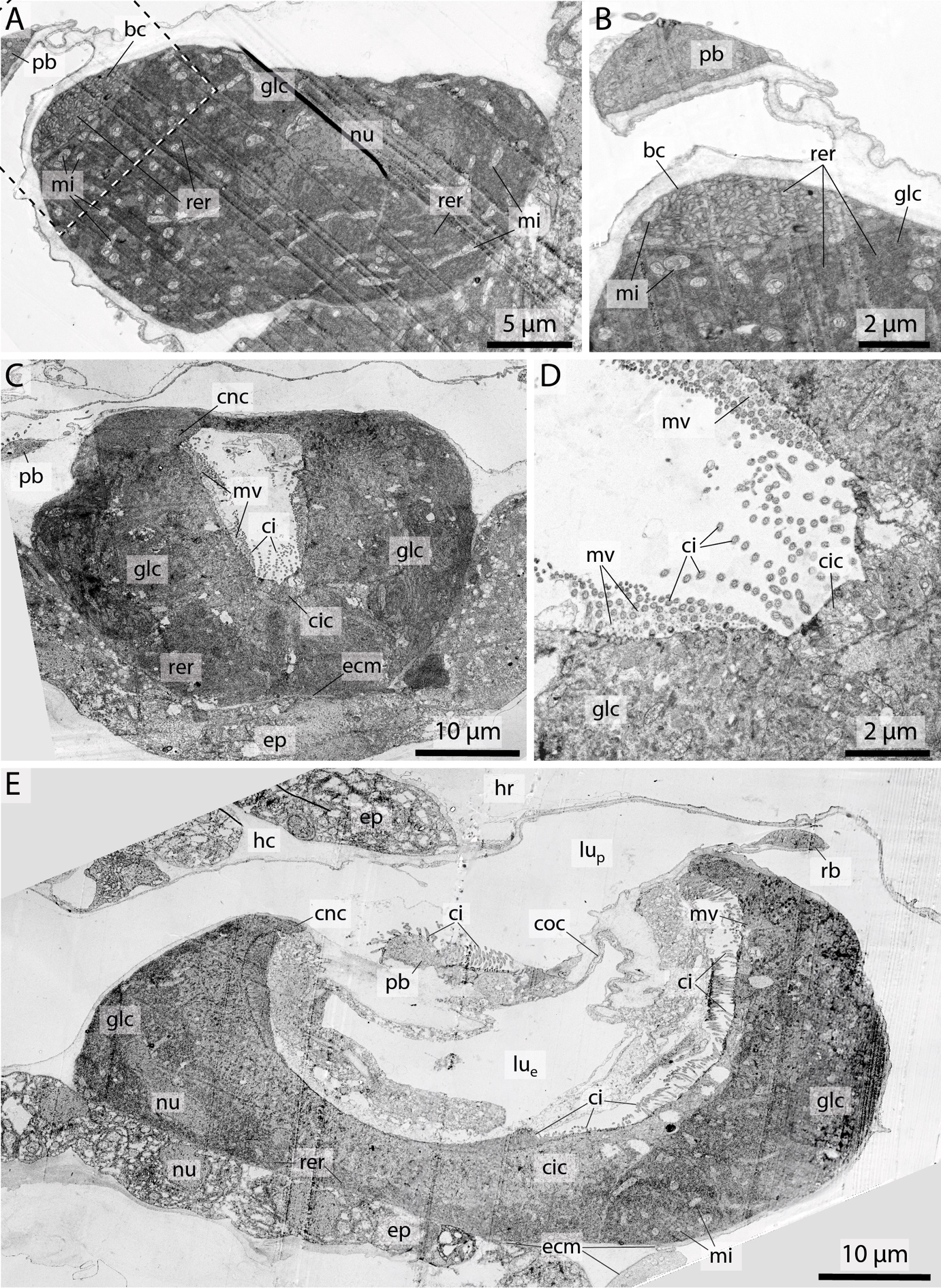


**Supplemental Figure S2.** Electron micrographs from the endostyle in *Fritillaria borealis.* **A**: Anterior to the left posterior to the right. Longitudinal section through the left side of the endostyle displaying gland cells (**glc**) with nuclei (**nu**) and an adjacent bright cell (**bc**) with less densely packed endoplasmic reticulum. Inset: light micrograph of an adjacent section showing the difference in toluidine staining between a gland cell and a bright cell. **B**: Closeup of area marked by dashed rectangle in A showing the bright cell as well as the adjacent gland cell with mitochondria (**mi**). Notice the different densities of endoplasmic reticulum. **C**: Cross section approximately between the center and the anterior end of the endostyle. Displaying the two paired rows of gland cells with ciliated cells (**cic**) nested at the ventral inner midline and their cilia (**ci**). **D**: Closeup of C displaying the arrangement of cilia in ciliated cells as well as the densely packed combination of cilia and microvilli from gland cells. E: Longitudinal section through the approximate center midline of the endostyle, displaying gland cells, ciliated cells with cilia as well as connecting cells (**cnc**) and corridor cells (**coc**).

**Supplemental Table T1**. Information on ultrathin and semithin sectioning for transmission electron microscopy (TEM) and light microscopy (LM) for each of the analyzed species as well as LM and TEM imaging magnifications.

| species | number of analysed specimens | first fixative | section thickness | total number of sections | used sections for reconstruction | magnification of light micrographs |
| --- | --- | --- | --- | --- | --- | --- |
| *M. huxleyi* | 1 | unknown, specimen from the natural history museum Berlin (likely also Glutaraldehyde) | 1 µm | 1605 | complete trunk - every 5th section (321), endostyle - every 2nd section (187) | complete trunk - 100x magnification, endostyle - 1000x magnification |
| *B. stygius* | 1 | 1% paraformaldehyde 2.5% glutaraldehyde in 0.2M sodium cacodylate buffer (pH 7.2) adjusted to an osmolarity of approximately 800 mOsm with added NaCl | 2 µm | 4250 | complete trunk - every 10th (425), endostyle - every section (276) | complete trunk - 25x magnification, endostyle - 200x magnification |
| *S. magnum* | 1 | 10% Formaldehyde | 1 µm | 2810 | complete trunk - every 10th (281), endostyle - every section (202) | complete trunk - 100x magnification, endostyle - 1000x magnification |
| *O. vanhoeffeni* | 1 | 10% Formaldehyde | 1 µm | 2030 | complete trunk- every 10th (203), endostyle- every 2nd (254) | complete trunk - 50x magnification, endostyle - 400x magnification |
| *O. dioica* | 2 | 1% paraformaldehyde 2.5% glutaraldehyde in 0.2M sodium cacodylate buffer (pH 7.2) adjusted to an osmolarity of approximately 800 mOsm with added NaCl | 1 µm | specimen 1: 291, specimen 2: 280 | specimen 1: complete trunk - every section (291), specimen 2: endostyle - every section (133) | complete trunk - 400x magnification, endostyle - 1000x magnification |
| *F. mediterra- nea* | 1 | 10% Formaldehyde | 1 µm | 972 | complete trunk - every 2nd section (486), endostyle - every section (176) | complete trunk - 400x magnification, endostyle - 1000x magnification |
| *F. pellucida* | 2 | 2% PFA and 2 % GA in PBS with adjusted NaCl (3%) | 0.5 µm | specimen 1: 3016, specimen 2: 1570 | specimen 1: complete trunk - every 8th section (377), endostyle - every 2nd section (247); specimen 2: endostyle - every 2nd section (257), not displayed in this study, used for confirmation) | complete trunk - 400x magnification, endostyle - 1000x magnification (for all specimens) |
| *F. borealis* | 3 | 2% PFA and 2 % GA in PBS with adjusted NaCl (3%) | 0.5 µm (LM), 70 nm (TEM) | specimen 1: 2144, specimen 2: 352, | specimen 1: complete trunk - every 8th section (171), endostyle (exemplary light micrographs, cross sections); specimen 2 : endostyle reconstruction - every section (112, LM, longitudinal); TEM (70 nm): exemplary sections (between every 16th light micrograph of specimen 1 and 2) | complete trunk - 400x magnification, endostyle - 1000x magnification (for specimen 1 and 2), TEM: 7000 x magnification (specimen 1 & 2) |
| *F. formica* | 1 | 10% Formaldehyde | 0.5 µm | 1728 | every 6th section (288) | complete trunk - 400x magnification |
| *F. halpostoma* | 1 | 10% Formaldehyde | 0.5 µm | 1135 | every 5th section (227) | complete trunk - 400x magnification |
| *K. oceanica* | 2 | 10% Formaldehyde | 0.5 µm | 980 | every 2nd section (490) | 400x magnification |
| *K. tenuis* | 1 | 10% Formaldehyde | 0.5 µm | 849 | every 3rd section (283) | 400x magnification |
